# Post-approval studies with the CFTR modulators Elexacaftor-Tezacaftor—Ivacaftor

**DOI:** 10.3389/fphar.2023.1158207

**Published:** 2023-03-21

**Authors:** Burkhard Tümmler

**Affiliations:** ^1^ Department of Pediatric Pneumology, Allergology and Neonatology, Hannover Medical School, Hannover, Germany; ^2^ Biomedical Research in Endstage and Obstructive Lung Disease Hannover (BREATH), German Center for Lung Research, Hannover Medical School, Hannover, Germany

**Keywords:** elexacaftor, tezacaftor, ivacaftor, CFTR, cystic fibrosis

## Abstract

Triple combination therapy with the CFTR modulators elexacaftor (ELX), tezacaftor (TEZ) and ivacaftor (IVA) has been qualified as a game changer in cystic fibrosis (CF). We provide an overview of the body of literature on ELX/TEZ/IVA published between November 2019 and February 2023 after approval by the regulators. Recombinant ELX/TEZ/IVA-bound Phe508del CFTR exhibits a wild type conformation *in vitro*, but in patient’s tissue a CFTR glyoisoform is synthesized that is distinct from the wild type and Phe508del isoforms. ELX/TEZ/IVA therapy improved the quality of life of people with CF in the real-life setting irrespective of their anthropometry and lung function at baseline. ELX/TEZ/IVA improved sinonasal and abdominal disease, lung function and morphology, airway microbiology and the basic defect of impaired epithelial chloride and bicarbonate transport. Pregnancy rates were increasing in women with CF. Side effects of mental status changes deserve particular attention in the future.

## Introduction

Cystic fibrosis (CF) is a severe ion channel disease of autosomal recessive inheritance that is caused by mutations in the *Cystic Fibrosis Transmembrane Conductance Regulator (CFTR)* gene. Thanks to continuously improved symptomatic treatment during the last 5 decades, this lethal paediatric disease has been transformed into a chronic disorder with a median life expectancy of nowadays more than 50 years (Bell et al., 2020).

Current therapy has been symptomatic, but meanwhile CFTR modulators have arrived to the clinic that target the basic defect in CF of impaired epithelial conductance for chloride and bicarbonate (Tümmler, 2022). There are two classes of CFTR modulators: Potentiators increase the activity of CFTR at the cell surface and correctors facilitate the translation, folding, maturation and trafficking of mutant CFTR to the cell surface and/or prevent its premature degradation. Already 10 years ago the potentiator ivacaftor has been approved for the treatment of the small group of patients who carry a gating mutation in at least one of their two *CFTR* alleles. Ivacaftor is the first molecule that has been approved as a mutation-type specific medication for human use. Meanwhile the triple combination of the potentiator ivacaftor (IVA) and the two correctors elexacaftor (ELX) and tezacaftor (TEZ) has become available for the treatment of the more than 90% of people with CF (pwCF) who harbour at least one *CFTR* allele that is responsive to this medication (Middleton et al., 2019). Thanks to the strong improvements in anthropometry, lung function, reduction of pulmonary exacerbations and quality of life, triple therapy with ELX/TEZ/IVA has been qualified as a game changer in CF (Bell et al., 2020). Based on an individual person-level microsimulation model the median lifetime survival of p.Phe508del homozygous pwCF receiving ELX/TEZ/IVA plus current best supportive care has been estimated to be 71.6 years (Lopez et al., 2023). ELX/TEZ/IVA is the first CFTR modulator therapy shown to halt lung function decline over an extended time period (Lee et al., 2022). This clinical success has initiated post-approval studies on multiple preclinical and clinical aspects. Here we now provide an overview of the current body of literature on ELX/TEZ/IVA published after approval in the United States by November 2019.

## CFTR modulators and their action on CFTR

Although there are more than 2,000 known sequence variants in *CFTR,* the vast majority of CF is homozygous or compound heterozygous for the most common mutation p.Phe508del. Phe508del CFTR protein is defective in posttranslational processing and trafficking. Newly synthesized Phe508del CFTR fails to adopt a wild-type fold in the endoplasmic reticulum (ER), is targeted to ER-associated degradation and is removed faster from the apical membrane by endocytosis. Consequently, p.Phe508del homozygous subjects express only low amounts of complex-glycosylated Phe508del CFTR and low or no residual Phe508del CFTR-mediated chloride and bicarbonate secretory activity. The analysis of second–site suppressor mutations revealed that a robust correction of the conformational defects of Phe508del CFTR requires the stabilization of the interfaces between the two nucleotide binding domains (NBDs) and the membrane-spanning domains (type I) and the stabilization of nucleotide binding domains 2 (NBD2) (type II) and Phe508del NBD1 (type III) (Okiyoneda et al., 2013). Combinations of type I, II and III correctors restored 50%–100% of wild-type-level Phe508del CFTR biogenesis and stability in immortalized and primary human airway epithelia (Veit et al., 2018). Concomitantly, the correctors decrease mucus concentration, relax mucus network ultrastructure, improve mucus transport and rheology of airway surface liquid, accelerate wound repair of the airway epithelium and change the plasma and cellular lipidome, in particular make the epithelial cells less susceptible to apoptosis by reducing the levels of ceramide (Gardner et al., 2020; Liessi et al., 2020; Veit et al., 2021a; Abu-Arish et al., 2022; Laselva and Conese, 2022; Ludovico et al., 2022; Morrison et al., 2022; Westhölter et al., 2022).

The yet most thoroughly characterized compound is the CFTR potentiator ivacaftor (IVA, VX-770, IUPAC name: N-(2,4-di-tert-butyl-5-hydroxyphenyl)-4-oxo-1,4-dihydroquinoline-3-carboxamide) (van Goor et al., 2009) (Table 1). The opening of the CFTR ion channel normally requires the binding and subsequent hydrolysis of ATP. In contrast, picomolar ivacaftor reversibly enhances ATP-independent opening of the channel and thereby overcomes the defective ATP-dependent opening of CF-causing gating mutations (Eckford et al., 2012; Jih and Hwang, 2013; Csanády and Töröcsik, 2019). CFTR open probability increases by stabilizing pre-hydrolytic states with respect to closed states (Kopeikin et al., 2014; Langron et al., 2018).

**TABLE 1:** Molecular action of the approved modulators on CFTR structure and function.

	Ivacaftor	Lumacaftor tezacaftor	Elexacaftor
Modulator type	Potentiator	Type I corrector	Type III corrector
Binding site in CFTR	ICL4 (photoaffinity labelling data) cleft formed by TM 4, 5, 8 (cryo-EM data)	TM 1, 2, 3, 6 (cryo-EM data)	TM 2, 10, 11, lasso motif (cryo-EM data)
Interaction with CFTR	stabilizes channel open configuration, enhances ATP-independent channel opening, stabilizes pre-hydrolytic states, reduces folding efficiency of Phe508del CFTR at the ER, destabilizes Phe508del CFTR in the plasma membrane	stabilizes early steps of CFTR biogenesis at the ER, improves co-translational folding of TMD1, facilitates binding of TMD1:NBD1 to ICL4, stabilizes the interactions NBD1:ICL4, NBD1:ICL1, TM3:TM4, TMD1:TMD2	supports assembly of the TMDs, co-potentiator

EM, electron micrograph; ICL, intracellular loop; NBD, nucleotide binding domain; TM, transmembrane helix; TMD, transmembrane domain.

Lumacaftor (LUM, VX-809, IUPAC name: 3-[6-[[[1-(2,2-difluoro-1,3-benzodioxol-5-yl) cyclopropyl]carbonyl] amino]- 3-methyl-2-pyridinyl]-benzoic acid) has been the first CFTR corrector approved for use in humans (van Goor et al., 2011). This type I corrector acts early during CFTR biosynthesis (Loo and Clarke, 2017; Kleizen et al., 2021) so that Phe508del CFTR can exit the ER (Table 1). It improves the co-translational folding of transmembrane domain 1 (TMD1). The subsequent early post-translational TMD1:NBD1 packing facilitates the most critical step of Phe508del CFTR folding, i.e., the binding to cytoplasmic loop 4 (ICL4), leading to progression of domain assembly in the absence of folded Phe508del-NBD1 (Kleizen et al., 2021). Further allosteric effects of lumacaftor are the stabilization of the NBD1:ICL4 and NBD1:ICL1 interfaces, of the transmembrane helices 3 and 4 and of the TMD1:TMD2 interaction (Farinha et al., 2013; He et al., 2013; Ren et al., 2013; Hudson et al., 2017; Loo and Clarke, 2017; Laselva et al., 2018; Krainer et al., 2020).

Tezacaftor (TEZ, VX-661, IUPAC name (R)-1-(2,2-difluorobenzo [d][1,3]dioxol-5-yl)-N-(1-(2,3-dihydroxypropyl)-6-fluoro-2-(1-hydroxy-2-methylpropan-2-yl)-1H-indol-5-yl) cyclopropane carboxamide) has been the second type I corrector that has been approved for the treatment of people with CF with one or two p.Phe508del alleles. Immunoblotting and *in silico* docking experiments proposed a similar composite multi-domain binding pocket for lumacaftor and tezacaftor comprised of residues within the NBD1:ICL4 interface (Molinski et al., 2018) (Table 1).

The type III corrector elexacaftor (ELX, VX-445, IUPAC name: *N*-(1,3-dimethylpyrazol-4-yl)sulfonyl-6-[3-(3,3,3-trifluoro-2,2-dimethylpropoxy)pyrazol-1-yl]-2-[(4*S*)-2,2,4-trimethylpyrrolidin-1-yl]pyridine-3-carboxamide) synergistically restores Phe508del CFTR processing in combination with type I or type II correctors (Veit et al., 2020; Becq et al., 2022) (Table 1). Moreover, elexacaftor acts as a co-potentiator of Phe508del, Gly551Asp and Met1101Lys CFTR chloride channels (Veit et al., 2021a; Laselva et al., 2021; Shaughnessy et al., 2021). Exposure of primary p.Phe508del homozygous epithelia to a triple combination of ELX/TEZ/IVA restored Phe508del CFTR chloride channel function to about 60% of wild-type levels (Veit et al., 2020; Capurro et al., 2021). However, when p.Phe508del homozygous cells were treated with ivacaftor combined to any correctors (LUM or TEZ or ELX), the Phe508del CFTR current was unresponsive to the subsequent acute addition of ivacaftor (Cholon et al., 2014; Veit et al., 2014; 2020; Shaughnessy et al., 2022a; Becq et al., 2022). Ivacaftor diminished the folding efficiency and the metabolic stability of Phe508 CFTR at the ER and post-ER compartments and destabilized rescued Phe508del CFTR at the plasma membrane causing reduced cell surface Phe508 CFTR density and function. CFTR Western blot analysis of intestinal epithelium of people with CF with one or two p.Phe508del alleles revealed that treatment with ELX/TEZ/IVA improves posttranslational processing and trafficking of Phe508del CFTR. However, a low-complexity Phe508del CFTR glycoisoform is produced that lacks the polydisperse spectrum of N-linked oligosaccharides of mature complex glycosylated wild type CFTR (Stanke et al., 2023). Hence, triple therapy with ELX/TEZ/IVA generates and stabilizes a novel Phe508del CFTR glycoisoform that is distinct from both the wild type and mutant isoforms.

Cryo-electron microscopy of reconstituted recombinant protein identified the binding sites of elexacaftor, tezacaftor and ivacaftor in wild type and Phe508del CFTR (Fiedorczuk & Chen, 2022). Clinically most relevant, the conformations of wild type CFTR and ELX/TEZ/IVA-bound Phe508del CFTR were almost indistinguishable from each other indicating that the CFTR modulators induce the “correct” conformation in the absence of any other members of the CFTR interactome. The three drugs bind to distinct sites of the CFTR protein described by Fiedorczuk and Chen (2022) as a “triangular belt encircling the transmembrane domains”. The potentiator ivacaftor binds to a cleft formed by transmembrane helices 4, 5, and 8 that stabilizes the open configuration of the ion pore in both wild type and Phe508del CFTR. Likewise, the type I corrector tezacaftor is recognized in both wild type and mutant by the same amino acid residues of transmembrane helices 1, 2, 3, and 6 and thereby probably stabilizes the early steps of CFTR biogenesis at the ER. Conversely, the type III corrector elexacaftor supports the subsequent assembly of the TMDs. ELX binds to Phe508del CFTR within the membrane mainly interacting with amino acid residues of transmembrane helices 2, 10, 11, and the N-terminal lasso motif.

## Clinical pharmacology

Published data on the pharmacokinetics of ELX/TEZ/IVA in humans are scarce and needs to be extracted from the material submitted by the manufacturer Vertex to the regulators (FDA or EMA). The serum half-life is 12 h for ivacaftor and 23 h for the correctors. Thus, the label recommends a morning dose with ELX/TEZ/IVA (TRIKAFTA®, KAFTRIO®) and an evening dose with IVA (Kalydeco®). A deuterated derivative of ivacaftor, called deutivacaftor (VX-561), has a reduced rate of clearance, greater plasma concentrations at 24 h, and a longer half-life compared with ivacaftor, thereby supporting once-daily dosing (Harbeson et al., 2017). Once-daily triple therapy of deutivacaftor together with tezacaftor and the novel corrector vanzacaftor is currently being examined in clinical trials (Uluer et al., 2023). Assays for quantifying ELX, TEZ, IVA in human plasma and cell lysate have meanwhile been established by academic labs applying multiple reaction monitoring mass spectrometry (MRM/MS) (Reyes-Ortega et al., 2020) or isotope dilution liquid chromatography tandem mass spectrometry (LC-MS/MS) (Habler et al., 2021; Ryan et al., 2022). Pharmacokinetic modelling revealed that the transition from IVA monotherapy or dual regimens with LUM/IVA or TEZ/IVA to triple combination therapy with ELX/TEZ/IVA will approach steady state levels within 2 weeks whereby IVA and at least one corrector will remain above the half-maximal effective concentration at all times (Tsai et al., 2020). Thus, although the individual CFTR modulators are differentially metabolized by the Cyp 3A457 enzyme complex in the liver, the immediate transition from mono or dual regimens to triple therapy seems to be safe.

A challenging problem is the treatment of pwCF who are infected with non-tuberculous mycobacteria (NTM), namely, *Mycobacterium abscessus, Mycobacterium bolleti* or *Mycobacterium avium.* Chronic airway infections with NTM have become the major risk factor for quality of life and prognosis in CF (Martiniano et al., 2022). The antimycobacterial multidrug regimens that are laborious and burdened with many side effects will include macrolide antibiotics and the ansamycins rifampicin or rifabutin. Marolides inhibit and ansamycins strongly induce the Cyp-system. Correspondingly, the label lists these drugs as contraindication for CFTR modulator therapy. Based on physiologically based pharmacokinetic modeling of drug-drug interaction, Hong et al. (2022) have recently proposed a dose-adjusted ELX/TEZ/IVA therapy concomitant with NTM treatment, i.e., increased doses of ELX/TEZ/IVA 200/100/450 mg in the morning and 100/50/375 mg in the evening when ETI is co-administered with rifabutin and reduced doses of ELX/TEZ/IVA 200/100/150 mg q72 h when co-administered with clofazimine or clarithromycin, respectively.

Safety data for the CFTR modulators show that therapy is well-tolerated. However, the phase 3 clinical studies for ELX/TEZ/IVA reported an incidence of rash ranging from 4% to 10.9%. Rash appeared to be more common in female patients and in those who use hormonal contraception. Meanwhile numerous adverse skin reactions have been observed in the real-life setting ranging from skin rash, drug-induced acne, eruptive melanocytic naevi to toxic epidermal necrolysis (Goldberg et al., 2021; Leonhardt et al., 2021; Atkinson et al., 2022; Bhaskaran and Bateman, 2022; Cheng et al., 2022; Diseroad et al., 2022; Hu et al., 2022; Hudson et al., 2022; Loyd et al., 2022; Mederos-Luis et al., 2022; Muirhead et al., 2022; Okroglic et al., 2023). Desensitization protocols were often successful, but etiology has not been examined, the exception being a case report of LUM-responsive CD4^+^ T-cell clones in non-immediate allergy (Roehmel et al., 2021).

## Impact of ELX/TEZ/IVA on CFTR biomarkers

Modulation of the basic defect in chloride and bicarbonate transport by ELX/TEZ/IVA has been assessed in post-approval studies in the sweat gland, kidney and the respiratory, biliary and intestinal epithelium of pwCF. CFTR-mediated chloride conductance of the upper respiratory epithelium improved to a mean 47% of normal with ELX/TEZ/IVA (Graeber et al., 2022a). Similarly, CFTR-mediated chloride transport of biliary and intestinal epithelium shifted into the normal range for most pwCF who carry at least one p.Phe508del allele (Graeber et al., 2022a; Bijvelds et al., 2022). In the kidney exposure to ELX/TEZ/IVA increased bicarbonate excretion to about 70% of that seen in healthy controls (Berg et al., 2022).

The responses of the sweat gland to ELX/TEZ/IVA were discordant. The sweat chloride concentration in the pilocarpine iontophoresis sweat test dropped by a mean 50 mmol/L into the intermediary or even normal range in the majority of pwCF implying that the basic defect of defective chloride reabsorption in the sweat duct had been partially or completely reversed by triple therapy (Graeber et al., 2022a). Conversely, the ß-adrenergic stimulated sweat secretion in the coil reached just about 5% of median wild type β-adrenergic sweat rate (Pallenberg et al., 2022a). Apparently β-adrenergic sweat stimulation in the coil is more stringent in its requirements for a wild type CFTR conformation whereas the reabsorption of chloride in the sweat duct tolerates residual structural and functional deficits of pharmacologically rescued mutant CFTR in the apical membrane. The limited response of the β-adrenergic sweat rate to high-efficient CFTR modulation allows the evaluation of new, potentially even more efficient CFTR modulators in the future, while the sweat chloride concentration may already have reached the limit of its sensitivity.

## Exposure of ELX/TEZ/IVA to rare *CFTR* genotypes

In Europe ELX/TEZ/IVA therapy is currently approved for pwCF aged 6 years or more who carry one or two p.Phe508del alleles. The label in the US just requests the carriage of at least one CF allele that is known to be responsive to the CFTR modulator *in vitro*. Thus, about 90% of pwCF have access to ELX/TEZ/IVA. A subgroup of the remaining 10% of the population is carrying rare or even ultra-rare mutations of unknown mutant phenotype. Thus, to address this unresolved issue, researchers have characterized the association between *CFTR* genotype, phenotype and its modulation by ELX/TEZ/IVA in recombinant cells (Laselva et al., 2021; Borgo et al., 2022; Tomati et al., 2022) or patient-derived epithelial cells *in vitro* (Veit et al., 2021b; Borgo et al., 2022; Shaughnessy et al., 2022b; Furstova et al., 2022; Tomati et al., 2022). Alternatively, they combined the cell culture work with the examination of CFTR biomarkers and clinical characteristics prior and during treatment with ELX/TEZ/IVA (Anderson et al., 2021; Comegna et al., 2021; Huang et al., 2021; Terlizzi et al., 2021; Aalbers et al., 2022; Kondratyeva et al., 2022a; 2022b; Ciciriello et al., 2022; Sondo et al., 2022). A peculiar challenge are complex alleles not yet documented in the databases. Characterization of p.[Leu467Phe-Phe508del] in patient-derived organoids and primary intestinal epithelium demonstrated a more compromised CFTR function than p.Phe508del, but fortunately was susceptible to modulation by ELX/TEZ/IVA both *in vitro* and in the patient *in vivo* (Kondratyeva et al., 2022a; Kondratyeva et al., 2022b).

Numerous cases with two non-Phe508del mutations yielded outcomes of triple therapy that would not have been expected from our knowledge of the molecular pathology of CFTR. Table 2 lists the published cases that by now have been examined prior to and during ELX/TEZ/IVA therapy with CFTR biomarkers. Table 3 provides data on sweat chloride, spirometry and body weight of pwCF with advanced lung disease who participated in the French Compassionate Program of ELX/TEZ/IVA (Burgel et al., 2023).

**TABLE 2:** Rare non-p.Phe508del *CFTR* genotypes assessed for ELX/TEZ/IVA—mediated CFTR modulation in pwCF by CFTR biomarkers. Sequence variants are differentiated by *CFTR* mutation class: class I, minimal function; class II, defective in protein processing and trafficking; class III: defective gating; class IV, change of ion channel conductance; class V, reduced amount of wild type CFTR.

CFTR genotype	CFTR biomarkers		References
class I—class I			
c.165-2 A>G/c.273 + 1G>A	SST:0.07/**0.23** QPIT: 84/102 NPD: 0/−3	ICM: 6/5	Pallenberg et al. (2023)
c.165-2 A>G/c.273 + 1G>A	SST:0.06/**0.23** QPIT: 110/115 NPD: 0/−1	ICM: 5/5	Pallenberg et al. (2023)
class I—class II			
p.Gly542Ter/p. [Leu467Phe-Phe508del]	ALI: 2/2		Sondo et al. (2022)
p.Glu585Ter/p. [Leu467Phe-Phe508del]	ALI: 5/5		Sondo et al. (2022)
p.Glu193Ter/p.Asn1303Lys	WPC: 0/**22** QPIT: 108/95		Huang et al. (2021)
class I—class III			
c.1585-1G>A/p.Gly1244Glu^a^	ALI: 1.7/**15**		Tomati et al. (2022)
p.Gly542Ter/p.Gly1244Glu^a^	ALI: 1.6/**16**		Tomati et al. (2022)
class III—class III			
p.Gly1244Glu*/p.Gly1244Glu^a^	ALI: 2.4/**20**		Tomati et al. (2022)

^a^ FDA approved sequence variant for ELX/TEZ/IVA, therapy.

Paired values at absence and presence of ELX/TEZ/IVA. A clinically relevant improvement of CFTR activity into the normal range or in the range of CFTR-related disorders is marked in bold.

SST, sweat secretion test: ß-adrenergically stimulated sweat secretion [nL/min].

QPIT, quantitative pilocarpin iontophoresis sweat test: sweat chloride concentration [mMol/L).

NPD, nasal transepithelial potential difference: cumulative depolarization potential to chloride-free solution [mV].

ICM, intestinal current measurement: cumulative ion current of rectal biopsy upon exposure to forskolin/IBMX and carbachol [µA/cm^2^].

ALI, transepithelial ion transport of primary nasal epithelial cells grown at air-liquid interface [µA/cm^2^].

WPC, whole patch-clamp recording of recombinant HEK293 cells [pA/pF].

**TABLE 3:** Rare non-p.Phe508del *CFTR* genotypes assessed for ELX/TEZ/IVA - mediated CFTR modulation by sweat test, spirometry and body weight in pwCF with advanced lung disease (Burgel et al., 2023). Sequence variants are differentiated by *CFTR* mutation class: class I, minimal function; class II, defective in protein processing and trafficking; class III: defective gating; class IV, change of ion channel conductance; class V, reduced amount of wild type CFTR.

*CFTR* genotype	Sweat chloride	ppFEV1	Body weight
	[mMol/L]	[% predicted]	[kg]
class I—class I			
*c.262-263delTT/p.Arg553Ter*	*107/92*	*44/42*	*36/36*
*c.357delC/c.357delC*	*70/96*	*28/28*	*59/56*
*c.579 + 1G>T/c.579 + 1G>T*	*110/96*	*42/44*	*28/29*
*c.948delT/p.Trp1282Ter*	*90/138*	*30/27*	*43/47*
c.1209G>A/c.2215delG	61/47	37/47	48/50
*c.1392G>T (p.Lys464Asn)/c.3528delC*	*94/95*	*23/21*	*47/47*
*c.1393-1G>A/c.1393-1G>A*	*86/80*	*42/45*	*39/41*
*c.1393-1G>A/c.1393-1G>A*	*93/89*	*41/46*	*68/70*
c.1585-1G>A/c.2051_2052delAAinsG	105/95	39/60	60/61
*c.1585-1G>A/c.3528delC*	*96/99*	*39/50*	*38/40*
*c.1585-1G>A/p.Gly542Ter*	*112/111*	*27/18*	*50/50*
*c.1585-1G>A/p.Arg553Ter*	*124/107*	*35/39*	*50/50*
*c.1679 + 1.6 kb A>G/c.1679 + 1.6 kb A>G*	*92/92*	*27/27*	*47/48*
*c.2051_2052delAAinsG/c.2051_2052delAAinsG*	*93/104*	*24/25*	*44/43*
*c.2051_2052delAAinsG/p.Gln493Ter*	*102/99*	*34/35*	*53/55*
*c.2051_2052delAAinsG/p.Gly542Ter*	*85/86*	*46/49*	*56/56*
*c.2810_2811insT/c.2989-313 A>T*	*98/65*	*32/29*	*55/55*
c.2909-1 T>G/c.2909-1 T>G	68/28	32/38	95/94
*c.2988 + 1G>A/c.2988 + 1G>A*	*106/102*	*31/32*	*60/59*
*c.2988 + 1G>A/c.2988 + 1G>A*	*111/124*	*26/26*	*30/30*
*c.2997_3000delAATT/p.Arg1162Ter*	*100/102*	*26/26*	*43/45*
*c.3469-2880_3717 + 2150del/c.3469-2880_3717 + 2150del*		*30/28*	*57/57*
*c.3964-3C>G/c.3964-3C>G*	*95/110*	*32/42*	*47/47*
*c.4139delC/p.Gly542Ter*	*102/102*	*37/44*	*22/23*
c.4242 + 1G>A/c.4242 + 1G>A	88/73	31/52	25/30
*c. [4242 + 1G>A; 3170delC]/p.Trp846Ter*	*101/95*	*40/37*	*49/48*
*p.Tyr122Ter/p.Tyr122Ter*	*114/111*		*49/47*
*p.Gly542Ter/p.Gly542Ter*	*103/98*	*35/33*	*58/58*
*p.Trp1063Ter/p.Trp1063Ter*	*120/110*	*27/28*	*65/65*
*p.Trp1282Ter/p.Trp1282Ter*	*108/97*	*28/27*	*62/62*
*p.Trp1282Ter/p.Trp1282Ter*	*114/111*	*18/11*	*36/36*
*p.Trp1282Ter/p.Trp1282Ter*	*104/101*	*28/29*	*59/59*
class I—class II			
CFTRdele2/p.Ala561Glu	100/61	22/42	61/63
c.489 + 2 T>G/p.Iso601Phe*	79/45	51/52	62/63
*c.579 + 1G>T/p.Iso507del*		*31/31*	*57/55*
*c.2051_2052delAAinsG/p.Leu558Ser*	*75/73*	*38/36*	*69/67*
c.2490 + 1G>A/p.Gly85Glu*	105/66	44/63	52/55
c.2805_2810delinsTCAGA/p.Arg1066Cys	(142)/70	23/38	62/67
c.3264delC/p.Met1101Lys*	(163)/45	37/66	36/38
p.Gln493Ter/p.Gly85Glu*	102/63	38/48	67/68
*p.Arg553Ter/p.Iso507del*		*25/30*	*49/49*
p.Glu585Ter/p.Arg1066Cys	100/56	25/40	33/34
p.Arg1162Ter/p.Asn1303Lys	99/90	32/61	32/33
p.Arg1162Ter/p.Asn1303Lys		23/34	51/55
p.Arg1162Ter/p.Asn1303Lys	(131)/95	46/54	51/52
class I—class IV			
c.579 + 1G>T/p. [Arg74Trp-Val201Met-Asp1270Asn]*	54/18	35/35	52/53
p.Trp1282Ter/p.Asp1152His*	38/30	43/49	69/67
class I—class V			
c.2051_2052delAAinsG/c.2657 + 5G>A	99/80	31/32	73/74
c.2988 + 1G>A/c.2657 + 5G>A		18/27	64/66
*c.2988 + 1G>A/c.2657 + 5G>A*	*97/87*	*28/31*	*51/50*
class II—class II			
p.Gly85Glu*/p.Gly85Glu*		24/32	75/79
p.Gly85Glu*/p.Gly85Glu*	96/76	46/60	57/60
p.Ser492Phe*/p.Arg1066Cys	73/28	34/41	54/56
p.His1085Arg*/p.Asn1303Lys	99/46	45/66	47/49
p.His1085Arg*/p.Asn1303Lys	97/23	29/62	46/49
p.Asn1303Lys/p.Asn1303Lys	109/87	19/30	50/53
p.Asn1303Lys/p.Asn1303Lys	105/96	33/92	57/62
p.Asn1303Lys/p.Asn1303Lys	93/92	44/69	41/44
p.Asn1303Lys/p.Asn1303Lys	114/76	23/32	54/56
p.Asn1303Lys/p.Asn1303Lys	96/91	20/30	61/63
class II—class IV			
p.Gly85Glu*/p.Arg334Trp	60/13	34/55	65/66
class IV—class IV			
p.Arg334Trp/p.Arg347Pro*	101/65	29/37	40/41
p.Arg347Pro*/p.Arg347Pro*	79/37	38/42	37/43
p.Arg347Pro*/p.Asn1303Lys	102/28	26/41	50/54
p.Ser364Pro*/p.Ser364Pro*	82/24	42/60	58/60
class V –class V			
*c.2657 + 5G>A/c.2657 + 5G>A*	*107/94*	*35/33*	*33/34*

^a^ FDA approved sequence variant for ELX/TEZ/IVA therapy.

Paired values at baseline and after at least 1 month of continuous treatment with ELX/TEZ/IVA; sweat chloride concentration [mMol/L] in QPIT; ppFEV1, percent of predicted Forced expiratory Volume in 1 s; body weight in kg. All individuals were modulator-naïve at baseline. Physiologically implausible sweat chloride concentrations are indicated by brackets.

Data were taken from Tables 2–4 compiled by the French Compassionate Program of the French CF reference study group (Burgel et al., 2023). The class of a mutation was allocated according to the molecular phenotype reported in the publications linked to the mutation in the CFTR1 database (http://www.genet.sickkids.on.ca/PicturePage.html). Sequence variants within canonical splice sites were assigned to class I.

PwCF, who were judged to be responders and continued therapy with ELX/TEZ/IVA, are indicated by normal font. PwCF, who were judged to be non-responders and discontinued therapy are indicated in italics.

The molecular phenotype of splice site mutations is typically predicted from the localization of the nucleotide substitution in the acceptor or donor splice sites. If an individual with CF carries a mutation in the canonical splice sites at the positions −2, −1, +1 or +2 at the intron/exon border, exon skipping will occur. The generated CFTR mRNA isoforms will typically be either rapidly degraded or translated into mutants of no or low activity. Thus, these splice mutations are assigned to the class I of minimal function. In line with expectation most class I/class I genotypes with one or two canonical splice site mutations did not respond to ELX/TEZ/IVA (Table 3). However, exceptions were noted. Triple therapy improved sweat chloride and lung function in pwCF who are homozygous for splice sites mutations affecting the inclusion of introns 18 and 26, respectively (Burgel et al., 2023). We tested two brothers who are compound heterozygous for an acceptor splice site and a donor splice site mutation flanking the same exon (Pallenberg et al., 2023). These index cases normalized CFTR function in the secretory coil of the sweat gland upon exposure to ELX/TEZ/IVA, whereas the respiratory and the intestinal epithelia were only slightly or not responsive to CFTR modulation (Table 2).

Class V splice mutations harbor the nucleotide substitution at a less conserved position of the splice site. Alternative splicing will generate both full-length and shorter CFTR mRNA isoforms. Hence, the donor splice mutation c.3717 + 5G>T has been expected to generate some wild type transcript associated with a pancreatic sufficient phenotype and a request for inclusion into the compassionate use program was denied. The index case, however, was exocrine pancreatic insufficient and the swelling assay in patient-derived intestinal organoids demonstrated a loss-of-function phenotype. The subject thus qualified for treatment with ELX/TEZ/IVA and then showed strong improvements in lung function, lung morphology and sweat chloride (Aalbers et al., 2022). On the other hand, the rather common splice mutation c.2657 + 5G>A is a class V mutation that is known to confer some residual wild-type CFTR activity (Highsmith et al., 1997; van Barneveld et al., 2008). However, ELX/TEZ/IVA therapy of pwCF with one or two c.2657 + 5G>A mutations led to only marginal or no clinical improvement (Table 3) (Burgel et al., 2023).

Asn1303Lys CFTR is post-translationally processed by other pathways than Phe508del CFTR. According to tests in recombinant cells Asn1303Lys CFTR was thought to be not responsive to CFTR modulation. However, ELX/TEZ/IVA efficiently attenuated the basic defect in numerous patients (Tables 2, 3) (Huang et al., 2021; Burgel et al., 2023). Similarly, the class II mutations p.Ala561Glu, p.Arg1066Cys and p.Met1101Lys that were non-responsive to CFTR correctors *in vitro*, were susceptible to CFTR modulation *in vivo*. ELX/TEZ/IVA significantly reduced sweat chloride and improved lung function (Table 3). Likewise, the missense mutants Arg334Trp and Arg347Pro CFTR have been judged to be not accessible to modulation because of their vicinity to the ion pore. However, the carriers of these class IV mutations showed a strong clinical benefit in sweat test, lung function and anthropometry.

In summary, the test of mutations in recombinant cells *in vitro* correctly predicted the response of pwCF to ELX/TEZ/IVA for most mutations, but was erroneous for a few splice and missense mutations. This experience demonstrates that the response of pwCF with ultra-rare *CFTR* mutations to ELX/TEZ/IVA should be tested by CFTR biomarkers and clinical characteristics. These probatory trials provide proper care for the patient and improve our knowledge of the molecular pathology of CFTR.

## Quality of life during ELX/TEZ/IVA therapy in patient groups not covered by phase 3 trials

The first approval of ELX/TEZ/IVA for human use was based on the outcome of phase 3 trials in pwCF aged 12 years or more with subnormal spirometry of 40%–90% FEV1 predicted. During the phase 3 trials ELX/TEZ/IVA treatment led to higher scores in all respiratory (Middleton et al., 2019) and non-respiratory domains (Fajac et al., 2022) of the Cystic Fibrosis Questionnaire-Revised, a validated measure of quality of life. Meanwhile we learnt that ELX/TEZ/IVA therapy improves the quality of life of pwCF irrespective of their anthropometry and lung function at baseline. Already after 4 months of triple therapy “patients generally reported a rapid impact on respiratory symptoms, sleep quality, general wellbeing and physical self-esteem, and a reduction in overall treatment burden. The majority of patients contrasted treatment burden, symptom severity, depression and a closed future marked by death or transplantation before ELX/TEZ/IVA, to renewed and unexpected physical strength, leading to greater self-confidence, autonomy and long-term planning, after treatment initiation” (Martin et al., 2021). Daily hospitalization and intravenous antibiotic rates were reported to decrease by 80% (Walter & Bass, 2022), which matches with the author’s experience at his CF clinic. Most encouragingly, 1-month treatment with ELX/TEZ/IVA improved ppFEV1 in pwCF with advanced lung disease by 11%–13% (Carnovale et al., 2021; Martin et al., 2022) leading to a pronounced decline in CF-related transplants by 55%–83% in CF centers in the US, France and Germany (Bermingham et al., 2021; Burgel et al., 2021; Ringshausen et al., 2023). Treatment burden decreased substantially in the need for intravenous antibiotics, oxygen therapy and non-invasive ventilation (Martin et al., 2022). Therapy with ELX/TEZ/IVA was safe and efficacious post liver transplant (McKinzie et al., 2022; Ragan et al., 2022). Conversely, when ELX/TEZ/IVA was prescribed to lung transplant recipients for extrapulmonary complications of CF, triple therapy was poorly tolerated with modest perceived extrapulmonary benefit so that about 40% of patients discontinued the medication (Doligalski et al., 2022; Ramos et al., 2022). In summary, with the exception of lung transplant recipients, treatment with ELX/TEZ/IVA led to a strong improvement of the quality of life.

## Sinonasal function

Most pwCF have chronic rhinosinusitis resulting in nasal obstruction, nasal polyposis, sinus infections, repeated surgeries and olfactory dysfunction. Independent of age and global disease severity, ELX/TEZ/IVA therapy led within a few days to clinically meaningful and persisting improvements in sinonasal quality of life as assessed by the SinoNasal Outcome Test (SNOT-22) (DiMango et al., 2021; Douglas et al., 2021; Beswick et al., 2022a; 2022b; Shakir et al., 2022; Stapleton et al., 2022; Bode et al., 2023; Castellanos et al., 2023). Nasal polyps decreased in size or even resolved. Sinus opacification and mucosal thickening improved on CT radiographs. However, quantitative olfactory function did not significantly change according to the Smell Identification Test (Beswick et al., 2022a).

## Pulmonary

The phase 3 trials reported a mean absolute increase in ppFEV1 of 14 points after 24 weeks of therapy with ELX/TEZ/IVA in pwCF with one p.Phe508del allele and a ppFEV1 of 40%–90% at baseline (Middleton et al., 2019). A similar absolute increase in the ppFEV1 of 15% was observed in French, Dutch, and Belgian CF patients with advanced pulmonary disease (ppFEV1< 40% at baseline) (Burgel et al., 2021; Kos et al., 2022; Stylemans et al., 2022). In a real-world, postapproval setting ELX/TEZ/IVA did not only significantly improve spirometry but also the lung clearance index as a measure of ventilation homogeneity (Graeber et al., 2022b; Stylemans et al., 2022). Air trapping, airway mucus plugging and bronchial wall thickening were reduced (Bec et al., 2022; Graeber et al., 2022b; FitzMaurice et al., 2022; Goralski et al., 2022; Fainardi et al., 2023). Likewise, functional MRI showed improvements in ventilation and perfusion (Streibel et al., 2023). During sleep the episodes of oxygen desaturation, apnea and hypopnea decreased in adult pwCF (Welsner et al., 2022; Giallongo et al., 2023).

## Immunology and airway microbiology

In CF lung disease, mucus stasis favors chronic colonization with opportunistic pathogens, which determines the quality of life and prognosis in most pwCF. In spite of improved antimicrobial therapies, the characteristic age-dependent sequence of initial dominance of *S. aureus* followed by chronic colonization with *P. aeruginosa* has remained largely unchanged during the last 50 years. Neither monotherapy with IVA nor dual LUM/IVA changed the infection epidemiology in CF, but ELX/TEZ/IVA initiation was associated with a rapid reduction in infection-related visits and antimicrobial use among pwCF (Miller et al., 2022). After 12-month of treatment with ELX/TEZ/IVA, the detection of *Staphylococcus aureus* and *Pseudomonas aeruginosa* decreased at single CF centers by 40% or more (Pallenberg et al., 2022b; Beck et al., 2023; Sheikh et al., 2023). Sputum microbiome diversity increased (Sosinski et al., 2022). Compared to pretreatment, the total bacterial load decreased, the individual species were more evenly distributed in the community, and the individual microbial metagenomes became more similar in their composition. However, the microbial network remained vulnerable to fragmentation. The initial shift of the CF airway microbiome was attributable to the ELX/TEZ/IVA-mediated gain of CFTR activity followed by a diversification driven by a group of commensals at the 1-year time point that are typical for healthy airways (Pallenberg et al., 2022b).

CFTR is not only present in the apical epithelial membrane, but it is also intracellularly detectable in professional phagocytes where it regulates pH and chloride homeostasis of the post-Golgi network. ELX/TEZ/IVA therapy improved chloride efflux and the phagocytic and bactericidal activities of CF monocytes (Zhang et al., 2022b; Cavinato et al., 2022; Gabillard-Lefort et al., 2022), reduced neutrophilic inflammation in the lung (De Vuyst et al., 2023), reduced systemic pro-inflammatory cytokines and normalized circulating immune cell composition (Sheikh et al., 2023) even in pwCF with advanced lung disease (Dhote et al., 2023).

## Intestine, pancreas, liver and nutrition

The phase 3 trials demonstrated a significant increase of BMI during ELX/TEZ/IVA therapy (Middleton et al., 2019). These improvements were confirmed in real-life settings. Parameters related to nutrient absorption such as weight, BMI, cholesterol and albumin were all significantly increased and the lipid profile improved independent of the diet composition (Carnovale et al., 2022; Petersen et al., 2022). Serum levels of fat-soluble vitamins increased (Wright et al., 2022; Francalanci et al., 2023) even leading to singular cases of hypervitaminosis (Miller and Foroozan, 2022; Wisnieweski et al., 2022). These findings call for adjustments in vitamin supplementation. ELX/TEZ/IVA attenuated abdominal pain, gastro-oesophageal reflux, poor appetite and disorders of bowel movement (Mainz et al., 2022). Fecal markers of inflammation decreased. Pancreatic insufficiency did not improve (Schwarzenberg et al., 2022).

CFTR is not expressed by the endocrine pancreas but fibrosis and CFTR dysfunction in the ducts trigger the emergence of diabetes as the major co-morbidity in CF. Studies on the impact of ELX/TEZ/IVA on glucose homeostasis yielded conflicting outcomes. Continuous glucose monitoring (CGM) and oral glucose tolerance tests (OGTT) did not detect any difference in glucose patterns after several months of ELX/TEZ/IVA therapy in three studies (Chan et al., 2022; Crow et al., 2022; Piona et al., 2022). In contrast, glucose patterns again assessed by CGM or OGTT improved in three other studies (Korten et al., 2022; Scully et al., 2022; Steinack et al., 2023). Thus, we still do not know whether or not ELX/TEZ/IVA ameliorate glucose homeostasis and/or any of its direct determinants.

Drug-induced liver injury is known as a potential side effect of the highly lipophilic CFTR modulators (Salehi et al., 2021; Lowry et al., 2022) and the mobilization of gall stones may cause biliary colic shortly after initiation with ELX/TEZ/IVA (Safirstein et al., 2021). Upon initiation of triple CFTR modulator therapy serum levels of bilirubin and liver transaminases will mildly increase after 3 months which is sustained but does not appear to increase further in the majority of pwCF (Tewkesbury et al., 2023). A recently published observational study reported that ELX/TEZ/IVA negatively affects liver stiffness and alters bile acid metabolism in children and adolescents (Schnell et al., 2023). Bile acid profiles revealed a decrease in unconjugated and an increase in glycine-conjugated derivatives. Share wave velocity derived by Acoustic Radiation Force Impulse Imaging (ARFI) increased in the younger patients which indicates an increase of liver stiffness known to correlate with liver fibrosis. Schnell and co-workers (2023) suggest that ARFI measurements and serum levels of glycine-conjugated bile acids could serve as early markers for liver deterioration during ELX/TEZ/IVA therapy.

## Reproductive tract and pregnancy

Most women with CF exhibit subfertility mainly driven by CFTR dysfunction that causes viscous cervical mucus presenting a physical barrier to sperm penetration. Thanks to the partial reversion of the basic defect and the globally improved health and prognosis, pregnancy rates are increasing in women with CF exposed to ELX/TEZ/IVA (Taylor-Cousar and Jain, 2021). According to two published case series (Kendle et al., 2021; O’Connor et al., 2021) females with CF achieved conception within a few weeks after initiating ELX/TEZ/IVA. Most women who discontinued ELX/TEZ/IVA during pregnancy out of concern for unknown fetal risk restarted because of clinical deterioration (Taylor-Cousar and Jain, 2021). Even a case of successful pregnancy and uncomplicated delivery has been reported for a woman with CF with very poor lung function (ppFEV1 23%) prior to conception (Balmpouzis et al., 2022).

ELX/TEZ/IVA pass the placental barrier (Collins et al., 2022). For example, a p.Phe508del homozygous infant was born who had been exposed to ELX/TEZ/IVA *in utero* from the p.Phe508del homozygous mother taking ELX/TEZ/IVA. The neonate presented with a false-negative neonatal CF screening test, normal pancreatic function and a borderline sweat chloride in sweat test indicating a partial reversion of the basic defect *in utero* (Fortner et al., 2021). Likewise, ELX/TEZ/IVA treatment of a p.Phe508del carrier who was pregnant with a p.Phe508del homozygous fetus, resolved a mid-gestation meconium ileus and led to the delivery of a child with normal pancreatic function and borderline sweat chloride in sweat test (Szentpetery et al., 2022).

On the other hand, recently one case of pulmonary hemorrhage and three cases of bilateral congenital cataracts were reported for infants who were exposed to ELX/TEZ/IVA *in utero* (Jain et al., 2022a; Nuytten et al., 2022).

Considering the limited data on the outcomes following CFTR modulator use during pregnancy and lactation, the MAYFLOWERS trial was initiated, which will examine the role of the continued use of modulators by comparing the pregnancy in women with CF who are modulator ineligible and in women with CF who choose to continue or discontinue CFTR modulator therapy during pregnancy and lactation (Jain et al., 2022b).

## Nervous system and psychosocial issues

CFTR is ubiquitously expressed in the central and peripheral nervous system during the fetal period and remains to be predominantly expressed along the hypothalamic–hypophyseal axis postnatally. PwCF are inconspicuous in their mental activities suggesting that the dysfunction or lack of CFTR in the brain is compensated by other ion channels. The phase 3 trials and the open-extension study did not find any neurologic or psychiatric side effects of ELX/TEZ/IVA therapy other than headache. Post approval, however, adverse events related to the nervous system have been reported. Patients complained about testicular or joint pain (Rotolo et al., 2020; Prajapati et al., 2021) or–more seriously–about substantial mental status changes (Zhang et al., 2022a; Heo et al., 2022; Spoletini et al., 2022; Arslan et al., 2023). Symptoms emerged within the first 3 months after initiating ELX/TEZ/IVA therapy (Heo et al., 2022). The six patients of the first case series described their symptoms as fogginess, slurred speech, short term memory loss, word finding difficulty or other mental status changes (Heo et al., 2022). Symptoms of insomnia decreased by changing morning and evening dose. Earlier this year Arslan and colleagues (2023) reported two adolescents with CF with new-onset depression and suicide attempts shortly after starting ELX/TEZ/IVA. In line with these case series, one out of five adults with CF seen at another CF center in the US initiated or changed a psychiatric medication (Zhang et al., 2022a). Of 266 CF adults who started ELX/TEZ/IVA, nineteen individuals reported deterioration in mental health with anxiety, low mood, insomnia and “brain fog” with reduced attention and concentration span, which impacted on day-to-day activity and quality of life (Spoletini et al., 2022). Dose adjustments monitored by lung function and sweat chloride, in conjunction with psychological support and prescription of antidepressants if indicated, attenuated or resolved the symptoms (Spoletini et al., 2022). The underlying mechanism responsible for this possible side effect of mental health remains unknown.

## Change of the symptom-oriented treatment program

The highly effective triple modulator therapy reduces numerous symptoms of typical CF disease and calls for changes of the symptom-oriented treatment program. Supplementation with pancreatic enzymes and fat-soluble vitamins needs to be adapted on a case-to-case basis as it has already been individually optimized in the pre-modulator era. If the absorption of nutrients and vitamins, particularly fat absorption, improves, the nutritional recommendations can switch from a calorie-rich diet to the balanced mixed diet of the healthy population. Many pwCF already change their therapy without consulting their professional CF team.

The SIMPLIFY consortium will examine in the next years if chronic therapies can be modified or even stopped (Mayer-Hamblett et al., 2021). Already within a few days of treatment, pwCF recognize a reduction of sputum production. Lung imaging demonstrated that intraluminal mucus plugging starts to be resolved (Graeber et al., 2022b). Hence, inhalation of mucolytics may become dispensable. The first SIMPLIFY study included two parallel, multicenter, open-label, randomized, controlled, non-inferiority trials at 80 participating clinics across the USA in the Cystic Fibrosis Therapeutics Development Network (Mayer-Hamblett et al., 2022). Study participants had an almost normal spirometry. Six-week discontinuation of daily inhalation DNase or hypertonic saline did not show any significant difference in the change of ppFEV1 when compared with continuing treatment.

## Open questions

ELX/TEZ/IVA has improved the quality of life and prognosis for pwCF. Table 4 summarizes our current knowledge of the response of pwCF to ELX/TEZ/IVA under real-life conditions. However, the 3 years since approval are too short to conclude whether triple modulator therapy may halt the progression of CF lung disease in the long-term. Domestic multicenter consortia like PROMISE (Nichols et al., 2021; 2022) will probably resolve this issue by stratifying the course of quality of life, anthropometry and airway disease depending on age and disease status when triple therapy was started. Own data of the microbial airway metagenome suggest that after intermittent normalization the dysbiosis was coming back after 1 year of ELX/TEZ/IVA therapy (Pallenberg et al., 2022b). Bacterial load of the airways is reduced during ELX/TEZ/IVA but the typical CF pathogens are only rarely eradicated. Hence, for the time being we have no clue whether or not antimicrobial chemotherapy needs to be continued with the same stringency. Likewise, considering the conflicting outcome of the published studies, the impact of CFTR modulator therapy on CF-related diabetes mellitus deserves to be further clarified. ELX/TEZ/IVA normalizes salt and water metabolism. Arterial blood pressure slightly increases which may put pwCF at the same risk for cardiovascular complications as the normal population (Gramegna et al., 2022). The probably under-reported side effects of mental status changes deserve particular attention. Future studies should tell us whether these disturbances of mental health reflect an inappropriate adaptation to the medication that changes the patient’s lifelong perspectives or whether–more likely—they are the inevitable consequence of the gain of CFTR function in the central nervous system that has never expressed functional CFTR before, but now has to cope with chloride channel activities that since conception had been fully compensated by other members of the neural network.

**TABLE 4:** Real-world response of pwCF to triple therapy with ELX/TEZ/IVA*.

Feature	Improvement	No improvement/side effect
General	quality of life (Cystic Fibrosis Questionnaire-Revised (CFQ-R) respiratory domain score)	minor or no improvement of extrapulmonary symptoms in lung transplant recipients
CFTR biomarkers	CFTR-mediated chloride reabsorption in sweat gland duct	CFTR-mediated sweat secretion of the secretory coil
	CFTR-mediated chloride conductance in respiratory epithelium	
	CFTR-mediated chloride secretion in intestinal epithelium	
	CFTR-mediated bicarbonate secretion in renal epithelium	
Sinonasal	SinoNasal Outcome Test (SNOT-22) of sinonasal quality of life	
	reduction of nasal polyposis, sinus opacification and mucosal thickening	
Lower airways	lung function improved (spirometry, multiple breath washout), reduction of mucus plugging, bronchial wall thickening, less pulmonary exacerbations, low or no sputum production	consolidations, perfusion defects invariant
Airway microbiology	reduction of bacterial load, lower detection rates of *S. aureus* and *P. aeruginosa*	microbial network remains fragile, still dysbiosis
Immunology	reduced systemic and lung inflammation	
Cardiovascular		rare: systemic arterial hypertension
Nutrition, intestine	increased absorption of nutrients and fat-soluble vitamins, weight gain	exocrine pancreatic insufficiency
CF-related diabetes	contradictory outcomes of post-approval studies	
Hepatobiliary system		bile acid metabolism; increase of liver stiffness in children and adolescents (?)
Dermatology		rare: skin rash, drurg-induced acne
Reproductive system	increase of pregnancy rate	
Mental health		mental status changes (5%—10% of adults)

*Information available from peer-reviewed original publications by 1 March 2023.

From the author’s point of view the major challenge in the future will probably be the patient’s adherence to treatment. The burden of the time-consuming symptom-oriented treatment programs needs to be reduced, but the improved prognosis should not get lost by man’s common attitude “you ought to, but you don’t.” Non-adherence is linked to poor health outcomes. Annual medication adherence to IVA that is as efficacious for pwCF with gating mutations as ELX/TEZ/IVA is for pwCF with one or two p.Phe508 alleles, has been extracted for the UK patient population from data of the national specialty pharmacy database (Mehta et al., 2021). The mean proportion of days covered by medication was 0.80. Clinical efficacy of treatment is high, and the medication is extremely expensive. Thus, at each clinic the CF team should join forces to ensure high rates of adherence in pwCF in the long run.

## References

[B1] Aalbers B. L. Brunsveld J. E. van der Ent C. K. van den Eijnden J. C. Beekman J. M. Heijerman H. G. M. (2022). Forskolin induced swelling (FIS) assay in intestinal organoids to guide eligibility for compassionate use treatment in a CF patient with a rare genotype. J. Cyst. Fibros. 21, 254–257. 10.1016/j.jcf.2022.01.008 35110005

[B2] Abu-Arish A. Pandžić E. Luo Y. Sato Y. Turner M. J. Wiseman P. W. (2022). Lipid-driven CFTR clustering is impaired in cystic fibrosis and restored by corrector drugs. J. Cell Sci. 135, jcs259002. 10.1242/jcs.259002 35060604 PMC8976878

[B3] Anderson J. D. Liu Z. Odom L. V. Kersh L. Guimbellot J. S. (2021). CFTR function and clinical response to modulators parallel nasal epithelial organoid swelling. Am. J. Physiol. Lung Cell. Mol. Physiol. 321, L119–L129. 10.1152/ajplung.00639.2020 34009038 PMC8321858

[B4] Arslan M. Chalmers S. Rentfrow K. Olson J. M. Dean V. Wylam M. E. (2023) Suicide attempts in adolescents with cystic fibrosis on Elexacaftor/Tezacaftor/Ivacaftor therapy. J. Cyst. Fibros.: S1569-1993(23) 00023–31. 10.1016/j.jcf.2023.01.015 36759252

[B5] Atkinson M. Johnson O. Wilson N. Walshaw M. FitzMaurice T. S. (2022). Eruptive melanocytic naevi following initiation of elexacaftor/ivacaftor/tezacaftor for cystic fibrosis. J. Cyst. Fibros. 21, 1070–1073. 10.1016/j.jcf.2022.06.004 35752560

[B6] Balmpouzis Z. Faure van Rossum A. Baud D. Panchaud A. Mitropoulou G. Mazza Stalder J. (2022). Successful pregnancy in a cystic fibrosis patient with a severe impairment of lung function receiving Elexacaftor-Tezacaftor-Ivacaftor. Respir. Med. Case Rep. 40, 101776. 10.1016/j.rmcr.2022.101776 36386288 PMC9664010

[B7] Bec R. Reynaud-Gaubert M. Arnaud F. Naud R. Dufeu N. Di Bisceglie M. (2022). Chest computed tomography improvement in patients with cystic fibrosis treated with elexacaftor-tezacaftor-ivacaftor: Early report. Eur. J. Radiol. 154, 110421. 10.1016/j.ejrad.2022.110421 35772339

[B8] Beck M. R. Hornick D. B. Pena T. A. Singh S. B. Wright B. A. (2023). Impact of elexacaftor/tezacaftor/ivacaftor on bacterial cultures from people with cystic fibrosis. Pediatr. Pulmonol. Epub ahead of print. 10.1002/ppul.26362 36807558

[B9] Becq F. Mirval S. Carrez T. Lévêque M. Billet A. Coraux C. (2022). The rescue of F508del-CFTR by elexacaftor/tezacaftor/ivacaftor (Trikafta) in human airway epithelial cells is underestimated due to the presence of ivacaftor. Eur. Respir. J. 59, 2100671. 10.1183/13993003.00671-2021 34266939

[B10] Bell S. C. Mall M. A. Gutierrez H. Macek M. Madge S. Davies J. C. (2020). The future of cystic fibrosis care: A global perspective. Lancet Respir. Med. 8, 65–124. 10.1016/S2213-2600(19)30337-6 31570318 PMC8862661

[B11] Berg P. Sorensen M. V. Rousing A. Q. Vebert Olesen H. Jensen-Fangel S. Jeppesen M. (2022). Challenged urine bicarbonate excretion as a measure of cystic fibrosis transmembrane conductance regulator function in cystic fibrosis. Ann. Intern. Med. 175, 1543–1551. 10.7326/M22-1741 36315944

[B12] Bermingham B. Rueschhoff A. Ratti G. Nesmith A. Goodwin D. Gray S. (2021). Short-term effect of elexacaftor-tezacaftor-ivacaftor on lung function and transplant planning in cystic fibrosis patients with advanced lung disease. J. Cyst. Fibros. 20, 768–771. 10.1016/j.jcf.2021.05.009 34162524

[B13] Beswick D. M. Humphries S. M. Balkissoon C. D. Strand M. Vladar E. K. Lynch D. A. (2022b). Impact of cystic fibrosis transmembrane conductance regulator therapy on chronic rhinosinusitis and health status: Deep learning CT analysis and patient-reported outcomes. Ann. Am. Thorac. Soc. 19, 12–19. 10.1513/AnnalsATS.202101-057OC 34436985 PMC8787790

[B14] Beswick D. M. Humphries S. M. Balkissoon C. D. Strand M. Vladar E. K. Ramakrishnan V. R. (2022a). Olfactory dysfunction in cystic fibrosis: Impact of CFTR modulator therapy. J. Cyst. Fibros. 21, e141–e147. 10.1016/j.jcf.2021.09.014 34598881

[B15] Bhaskaran D. Bateman K. (2022). A case of Elexacaftor-Tezacaftor-Ivacaftor induced rash resolving without interruption of treatment. J. Cyst. Fibros. 21, 1077–1079. 10.1016/j.jcf.2022.06.011 35840534

[B16] Bijvelds M. J. C. Roos F. J. M. Meijsen K. F. Roest H. P. Verstegen M. M. A. Janssens H. M. (2022). Rescue of chloride and bicarbonate transport by elexacaftor-ivacaftor-tezacaftor in organoid-derived CF intestinal and cholangiocyte monolayers. J. Cyst. Fibros. 21, 537–543. 10.1016/j.jcf.2021.12.006 34922851

[B17] Bode S. F. N. Rapp H. Lienert N. Appel H. Fabricius D. (2023). Effects of CFTR-modulator triple therapy on sinunasal symptoms in children and adults with cystic fibrosis. Eur. Arch. Otorhinolaryngol. Epub ahead of print. 10.1007/s00405-023-07859-4 PMC10219879 36738326

[B18] Borgo C. D'Amore C. Capurro V. Tomati V. Sondo E. Cresta F. (2022). Targeting the E1 ubiquitin-activating enzyme (UBA1) improves elexacaftor/tezacaftor/ivacaftor efficacy towards F508del and rare misfolded CFTR mutants. Cell. Mol. Life Sci. 79, 192. 10.1007/s00018-022-04215-3 35292885 PMC8924136

[B19] Burgel P. R. Durieu I. Chiron R. Ramel S. Danner-Boucher I. Prevotat A. (2021). Rapid improvement after starting elexacaftor-tezacaftor-ivacaftor in patients with cystic fibrosis and advanced pulmonary disease. Am. J. Respir. Crit. Care Med. 204, 64–73. 10.1164/rccm.202011-4153OC 33600738

[B20] Burgel P. R. Sermet-Gaudelus I. Durieu I. Kanaan R. Macey J. Grenet D. (2023). The French Compassionate Program of elexacaftor-tezacaftor-ivacaftor in people with cystic fibrosis with advanced lung disease and no F508del CFTR variant. Eur. Respir. J. 2023, 2202437. Online ahead of print. 10.1183/13993003.02437-2022 36796836

[B21] Capurro V. Tomati V. Sondo E. Renda M. Borrelli A. Pastorino C. (2021). Partial rescue of F508del-CFTR stability and trafficking defects by double corrector treatment. Int. J. Mol. Sci. 22, 5262. 10.3390/ijms22105262 34067708 PMC8156943

[B22] Carnovale V. Iacotucci P. Terlizzi V. Colangelo C. Medio P. Ferrillo L. (2021). Effectiveness and safety of elexacaftor/tezacaftor/ivacaftor in patients with cystic fibrosis and advanced lung disease with the Phe508del/minimal function genotype. Respir. Med. 189, 106646. 10.1016/j.rmed.2021.106646 34673344

[B23] Carnovale V. Scialò F. Gelzo M. Iacotucci P. Amato F. Zarrilli F. (2022). Cystic Fibrosis Patients with F508del/Minimal Function Genotype: Laboratory and Nutritional Evaluations after One Year of Elexacaftor/Tezacaftor/Ivacaftor Treatment. J. Clin. Med. 11, 6900. 10.3390/jcm11236900 36498475 PMC9735556

[B24] Castellanos C. X. Osterbauer B. Hasday S. Keens T. G. Koempel J. Ference E. H. (2023). Improvement in sinonasal quality-of-life indicators for pediatric patients with cystic fibrosis treated with elexacaftor-tezacaftor-ivacaftor. Int. Forum Allergy Rhinol. 13, 72–75. 10.1002/alr.23036 35643960

[B25] Cavinato L. Luly F. R. Pastore V. Chiappetta D. Sangiorgi G. Ferrara E. (2022). Elexacaftor-Tezacaftor-Ivacaftor corrects monocyte microbicidal deficiency in cystic fibrosis. Eur. Respir. J. 2022, 2200725. Epub ahead of print. 10.1183/13993003.00725-2022 PMC10066567 36455959

[B26] Chan C. L. Granados A. Moheet A. Singh S. Vigers T. Arbeláez A. M. (2022). Glycemia and β-cell function before and after elexacaftor/tezacaftor/ivacaftor in youth and adults with cystic fibrosis. J. Clin. Transl. Endocrinol. 30, 100311. 10.1016/j.jcte.2022.100311 36620757 PMC9816065

[B27] Cheng A. Baker O. Hill U. (2022). Elexacaftor, tezacaftor and ivacaftor: A case of severe rash and approach to desensitisation. BMJ Case Rep. 15, e247042. 10.1136/bcr-2021-247042 PMC8895886 35236685

[B28] Cholon D. M. Quinney N. L. Fulcher M. L. Esther C. R. Jr. Das J. Dokholyan N. V. (2014). Potentiator ivacaftor abrogates pharmacological correction of ΔF508 CFTR in cystic fibrosis. Sci. Transl. Med. 6, 246ra96. 10.1126/scitranslmed.3008680 PMC4272825 25101886

[B29] Ciciriello F. Bijvelds M. J. C. Alghisi F. Meijsen K. F. Cristiani L. Sorio C. (2022). Theratyping of the rare CFTR variants E193K and R334W in rectal organoid-derived epithelial monolayers. J. Pers. Med. 12, 632. 10.3390/jpm12040632 35455747 PMC9027586

[B30] Collins B. Fortner C. Cotey A. Esther C. R. Jr. Trimble A. (2022). Drug exposure to infants born to mothers taking Elexacaftor, Tezacaftor, and Ivacaftor. J. Cyst. Fibros. 21, 725–727. 10.1016/j.jcf.2021.12.004 34952795 PMC9213569

[B31] Comegna M. Terlizzi V. Salvatore D. Colangelo C. Di Lullo A. M. Zollo I. (2021). Elexacaftor-Tezacaftor-Ivacaftor Therapy for Cystic Fibrosis Patients with the F508del/Unknown Genotype. Antibiot. (Basel) 10, 828. 10.3390/antibiotics10070828 PMC8300667 34356748

[B32] Crow H. Bengtson C. Shi X. Graves L. 3rd. Anabtawi A. (2022). CGM patterns in adults with cystic fibrosis-related diabetes before and after elexacaftor-tezacaftor-ivacaftor therapy. J. Clin. Transl. Endocrinol. 30, 100307. 10.1016/j.jcte.2022.100307 36217440 PMC9547287

[B33] Csanády L. Töröcsik B. (2019). Cystic fibrosis drug ivacaftor stimulates CFTR channels at picomolar concentrations. Elife 8, e46450. 10.7554/eLife.46450 31205003 PMC6594753

[B34] De Vuyst R. C. Bennard E. Kam C. W. McKinzie C. J. Esther C. R. (2023). Elexacaftor/tezacaftor/ivacaftor treatment reduces airway inflammation in cystic fibrosis. Pediatr. Pulmonol. 2023, 26334. 10.1002/ppul.26334 36718851

[B35] Dhote T. Martin C. Regard L. Pesenti L. Kanaan R. Carlier N. (2023). Normalisation of circulating neutrophil counts after 12 months of elexacaftor-tezacaftor-ivacaftor in patients with advanced cystic fibrosis. Eur. Respir. J. 61, 2202096. 10.1183/13993003.02096-2022 36455960

[B36] DiMango E. Overdevest J. Keating C. Francis S. F. Dansky D. Gudis D. (2021). Effect of highly effective modulator treatment on sinonasal symptoms in cystic fibrosis. J. Cyst. Fibros. 20, 460–463. 10.1016/j.jcf.2020.07.002 32694034

[B37] Diseroad E. R. Mogayzel P. J. Jr. Pan A. (2022). Rechallenge of elexacaftor/tezacaftor/ivacaftor after skin rash in two pediatric patients. J. Pediatr. Pharmacol. Ther. 27, 463–466. 10.5863/1551-6776-27.5.463 35845562 PMC9268115

[B38] Doligalski C. T. McKinzie C. J. Yang A. Lobo L. J. Coakley R. (2022). Poor tolerability of cystic fibrosis transmembrane conductance regulator modulator therapy in lung transplant recipients. Pharmacotherapy 42, 580–584. 10.1002/phar.2710 35689451

[B39] Douglas J. E. Civantos A. M. Locke T. B. Sweis A. M. Hadjiliadis D. Hong G. (2021). Impact of novel CFTR modulator on sinonasal quality of life in adult patients with cystic fibrosis. Int. Forum Allergy Rhinol. 11, 201–203. 10.1002/alr.22716 33070454

[B40] Eckford P. D. Li C. Ramjeesingh M. Bear C. E. (2012). Cystic fibrosis transmembrane conductance regulator (CFTR) potentiator VX-770 (ivacaftor) opens the defective channel gate of mutant CFTR in a phosphorylation-dependent but ATP-independent manner. J. Biol. Chem. 287, 36639–36649. 10.1074/jbc.M112.393637 22942289 PMC3481266

[B41] Fainardi V. Skenderaj K. Ciuni A. Esposito S. Sverzellati N. Pisi G. (2023). Effect of elexacaftor-tezacaftor-ivacaftor modulator on lung structure in cystic fibrosis. Pulmonology S2531-0437 (22), 00281–1. Epub ahead of print. 10.1016/j.pulmoe.2022.11.007 36564238

[B42] Fajac I. Daines C. Durieu I. Goralski J. L. Heijerman H. Knoop C. (2022). Non-respiratory health-related quality of life in people with cystic fibrosis receiving elexacaftor/tezacaftor/ivacaftor. J. Cyst. Fibros.:S1569-1993(22) 00655–5. 10.1016/j.jcf.2022.08.018 36114142

[B43] Farinha C. M. King-Underwood J. Sousa M. Correia A. R. Henriques B. J. Roxo-Rosa M. (2013). Revertants, low temperature, and correctors reveal the mechanism of F508del-CFTR rescue by VX-809 and suggest multiple agents for full correction. Chem. Biol. 20, 943–955. 10.1016/j.chembiol.2013.06.004 23890012

[B44] Fiedorczuk K. Chen J. (2022). Molecular structures reveal synergistic rescue of Δ508 CFTR by Trikafta modulators. Science 378, 284–290. 10.1126/science.ade2216 36264792 PMC9912939

[B45] FitzMaurice T. S. McCann C. Nazareth D. Shaw M. McNamara P. S. Walshaw M. J. (2022). Measuring the effect of elexacaftor/tezacaftor/ivacaftor combination therapy on the respiratory pump in people with CF using dynamic chest radiography. J. Cyst. Fibros. 21, 1036–1041. 10.1016/j.jcf.2022.01.007 35101365

[B46] Fortner C. N. Seguin J. M. Kay D. M. (2021). Normal pancreatic function and false-negative CF newborn screen in a child born to a mother taking CFTR modulator therapy during pregnancy. J. Cyst. Fibros. 20, 835–836. 10.1016/j.jcf.2021.03.018 33846105

[B47] Francalanci M. Terlizzi V. Fevola C. Di Rosa G. Pierattini V. Roselli E. (2023). Nutritional status and circulating levels of fat-soluble vitamins in cystic fibrosis patients: A cohort study and evaluation of the effect of CFTR modulators. Child. (Basel). 10, 252. 10.3390/children10020252 PMC9955178 36832382

[B48] Furstova E. Dousova T. Beranek J. Libik M. Fila L. Modrak M. (2022). Response to elexacaftor/tezacaftor/ivacaftor in intestinal organoids derived from people with cystic fibrosis. J. Cyst. Fibros. 21, 243–245. 10.1016/j.jcf.2021.07.006 34348870

[B49] Gabillard-Lefort C. Casey M. Glasgow A. M. A. Boland F. Kerr O. Marron E. (2022). Trikafta rescues CFTR and lowers monocyte P2X7R-induced inflammasome activation in cystic fibrosis. Am. J. Respir. Crit. Care Med. 205, 783–794. 10.1164/rccm.202106-1426OC 35021019

[B50] Gardner A. I. Haq I. J. Simpson A. J. Becker K. A. Gallagher J. Saint-Criq V. (2020). Recombinant acid ceramidase reduces inflammation and infection in cystic fibrosis. Am. J. Respir. Crit. Care Med. 202, 1133–1145. 10.1164/rccm.202001-0180OC 32569477 PMC7560813

[B51] Giallongo A. Parisi G. F. Papale M. Manti S. Mulé E. Aloisio D. (2023). Effects of elexacaftor/tezacaftor/ivacaftor on cardiorespiratory polygraphy parameters and respiratory muscle strength in cystic fibrosis patients with severe lung disease. Genes (Basel). 14, 449. 10.3390/genes14020449 36833376 PMC9956139

[B52] Goldberg R. H. Matthews N. H. Hristov A. C. Wang F. (2021). Urticaria multiforme-like eruption due to a novel agent elexacaftor/tezacaftor/ivacaftor in a pediatric patient with cystic fibrosis. JAAD Case Rep. 18, 71–73. 10.1016/j.jdcr.2021.10.018 34841028 PMC8606295

[B53] Goralski J. L. Chung S. H. Ceppe A. S. Powell M. Z. Sakthivel M. Handly B. D. (2022). Dynamic perfluorinated gas MRI shows improved lung ventilation in people with cystic fibrosis after elexacaftor/tezacaftor/ivacaftor: An observational study. J. Clin. Med. 11, 6160. 10.3390/jcm11206160 36294480 PMC9604637

[B54] Graeber S. Y. Renz D. M. Stahl M. Pallenberg S. T. Sommerburg O. Naehrlich L. (2022b). Effects of Elexacaftor/Tezacaftor/Ivacaftor Therapy on Lung Clearance Index and Magnetic Resonance Imaging in Patients with Cystic Fibrosis and One or Two F508del Alleles. Am. J. Respir. Crit. Care Med. 206, 311–320. 10.1164/rccm.202201-0219OC 35536314

[B55] Graeber S. Y. Vitzthum C. Pallenberg S. T. Naehrlich L. Stahl M. Rohrbach A. (2022a). Effects of Elexacaftor/Tezacaftor/Ivacaftor Therapy on CFTR Function in Patients with Cystic Fibrosis and One or Two *F508del* Alleles. Am. J. Respir. Crit. Care Med. 205, 540–549. 10.1164/rccm.202110-2249OC 34936849

[B56] Gramegna A. De Petro C. Leonardi G. Contarini M. Amati F. Meazza R. (2022). Onset of systemic arterial hypertension after initiation of elexacaftor/tezacaftor/ivacaftor in adults with cystic fibrosis: A case series. J. Cyst. Fibros. 21, 885–887. 10.1016/j.jcf.2022.04.010 35450770

[B57] Habler K. Kalla A. S. Rychlik M. Bruegel M. Teupser D. Nährig S. (2021). Isotope dilution LC-MS/MS quantification of the cystic fibrosis transmembrane conductance regulator (CFTR) modulators ivacaftor, lumacaftor, tezacaftor, elexacaftor, and their major metabolites in human serum. Clin. Chem. Lab. Med. 60, 82–91. 10.1515/cclm-2021-0724 34668357

[B58] Harbeson S. L. Morgan A. J. Liu J. F. Aslanian A. M. Nguyen S. Bridson G. W. (2017). Altering metabolic profiles of drugs by precision deuteration 2: Discovery of a deuterated analog of ivacaftor with differentiated pharmacokinetics for clinical development. J. Pharmacol. Exp. Ther. 362, 359–367. 10.1124/jpet.117.241497 28611092

[B59] He L. Kota P. Aleksandrov A. A. Cui L. Jensen T. Dokholyan N. V. (2013). Correctors of ΔF508 CFTR restore global conformational maturation without thermally stabilizing the mutant protein. FASEB J. 27, 536–545. 10.1096/fj.12-216119 23104983 PMC3545534

[B60] Heo S. Young D. C. Safirstein J. Bourque B. Antell M. H. Diloreto S. (2022). Mental status changes during elexacaftor/tezacaftor/ivacaftor therapy. J. Cyst. Fibros. 21, 339–343. 10.1016/j.jcf.2021.10.002 34742667

[B61] Highsmith W. E. Jr. Burch L. H. Zhou Z. Olsen J. C. Strong T. V. Smith T. (1997) Identification of a splice site mutation (2789 +5 G > A) associated with small amounts of normal CFTR mRNA and mild cystic fibrosis. Hum. Mutat. 9, 332–338. 10.1002/(SICI)1098-1004(1997)9:4<332::AID-HUMU5>3.0.CO 9101293

[B62] Hong E. Almond L. M. Chung P. S. Rao A. P. Beringer P. M. (2022). Physiologically based pharmacokinetic modeling to guide management of drug interactions between elexacaftor-tezacaftor-ivacaftor and antibiotics for the treatment of nontuberculous mycobacteria. Antimicrob. Agents Chemother. 66, e0110422. 10.1128/aac.01104-22 36286508 PMC9664863

[B63] Hu M. K. Wood G. Dempsey O. (2022). Triple therapy' (elexacaftor, tezacaftor, ivacaftor) skin rash in patients with cystic fibrosis. Postgrad. Med. J. 98, 86. 10.1136/postgradmedj-2020-139264 33310895

[B64] Huang Y. Paul G. Lee J. Yarlagadda S. McCoy K. Naren A. P. (2021). Elexacaftor/tezacaftor/ivacaftor improved clinical outcomes in a patient with N1303K-CFTR based on *in vitro* experimental evidence. Am. J. Respir. Crit. Care Med. 204, 1231–1235. 10.1164/rccm.202101-0090LE 34379998 PMC8759307

[B65] Hudson B. N. Jacobs H. R. Philbrick A. Zhou X. A. Simonsen M. M. Safirstein J. A. (2022). Drug-induced acne with elexacaftor/tezacaftor/ivacaftor in people with cystic fibrosis. J. Cyst. Fibros. 21, 1066–1069. 10.1016/j.jcf.2022.09.002 36088208

[B66] Hudson R. P. Dawson J. E. Chong P. A. Yang Z. Millen L. Thomas P. J. (2017). Direct binding of the corrector VX-809 to human CFTR NBD1: Evidence of an allosteric coupling between the binding site and the NBD1:CL4 interface. Mol. Pharmacol. 92, 124–135. 10.1124/mol.117.108373 28546419

[B67] Jain R. Magaret A. Vu P. T. VanDalfsen J. M. Keller A. Wilson A. (2022b). Prospectively evaluating maternal and fetal outcomes in the era of CFTR modulators: The MAYFLOWERS observational clinical trial study design. BMJ Open Respir. Res. 9, e001289. 10.1136/bmjresp-2022-001289 PMC9204448 35710144

[B68] Jain R. Wolf A. Molad M. Taylor-Cousar J. Esther C. R. Jr. Shteinberg M. (2022a). Congenital bilateral cataracts in newborns exposed to elexacaftor-tezacaftor-ivacaftor *in utero* and while breast feeding. J. Cyst. Fibros. 21, 1074–1076. 10.1016/j.jcf.2022.10.004 36266182

[B69] Jih K. Y. Hwang T. C. (2013). Vx-770 potentiates CFTR function by promoting decoupling between the gating cycle and ATP hydrolysis cycle. Proc. Natl. Acad. Sci. U. S. A. 110, 4404–4409. 10.1073/pnas.1215982110 23440202 PMC3600496

[B70] Kendle A. M. Roekner J. T. Santillana E. C. Kis L. E. Cain M. A. (2021). Cystic fibrosis transmembrane conductance regulator modulators during pregnancy: A case series. Cureus 13, e17427. 10.7759/cureus.17427 34589336 PMC8460487

[B71] Kleizen B. van Willigen M. Mijnders M. Peters F. Grudniewska M. Hillenaar T. (2021). Co-translational folding of the first transmembrane domain of ABC-transporter CFTR is supported by assembly with the first cytosolic domain. J. Mol. Biol. 433, 166955. 10.1016/j.jmb.2021.166955 33771570

[B72] Kondratyeva E. Bulatenko N. Melyanovskaya Y. Efremova A. Zhekaite E. Sherman V. (2022a). Personalized Selection of a CFTR Modulator for a Patient with a Complex Allele [L467F;F508del]. Curr. Issues Mol. Biol. 44, 5126–5138. 10.3390/cimb44100349 36286063 PMC9600521

[B73] Kondratyeva E. Efremova A. Melyanovskaya Y. Voronkova A. Polyakov A. Bulatenko N. (2022b). Evaluation of the Complex p.[Leu467Phe;Phe508del] CFTR Allele in the Intestinal Organoids Model: Implications for Therapy. Int. J. Mol. Sci. 23, 10377. 10.3390/ijms231810377 36142302 PMC9499621

[B74] Kopeikin Z. Yuksek Z. Yang H. Y. Bompadre S. G. (2014). Combined effects of VX-770 and VX-809 on several functional abnormalities of F508del-CFTR channels. J. Cyst. Fibros. 13, 508–514. 10.1016/j.jcf.2014.04.003 24796242

[B75] Korten I. Kieninger E. Krueger L. Bullo M. Flück C. E. Latzin P. (2022). Short-term effects of elexacaftor/tezacaftor/ivacaftor combination on glucose tolerance in young people with cystic fibrosis-an observational pilot study. Front. Pediatr. 10, 852551. 10.3389/fped.2022.852551 35529332 PMC9070552

[B76] Kos R. Neerincx A. H. Fenn D. W. Brinkman P. Lub R. Vonk S. E. M. (2022). Real-life efficacy and safety of elexacaftor/tezacaftor/ivacaftor on severe cystic fibrosis lung disease patients. Pharmacol. Res. Perspect. 10, e01015. 10.1002/prp2.1015 36440690 PMC9703582

[B77] Krainer G. Schenkel M. Hartmann A. Ravamehr-Lake D. Deber C. M. Schlierf M. (2020). CFTR transmembrane segments are impaired in their conformational adaptability by a pathogenic loop mutation and dynamically stabilized by Lumacaftor. J. Biol. Chem. 295, 1985–1991. 10.1074/jbc.AC119.011360 31882543 PMC7029128

[B78] Langron E. Prins S. Vergani P. (2018). Potentiation of the cystic fibrosis transmembrane conductance regulator by VX-770 involves stabilization of the pre-hydrolytic O_1_ state. Br. J. Pharmacol. 175, 3990–4002. 10.1111/bph.14475 30107029 PMC6151340

[B79] Laselva O. Ardelean M. C. Bear C. E. (2021). Phenotyping rare CFTR mutations reveal functional expression defects restored by TRIKAFTA^TM^ . J. Pers. Med. 11, 301. 10.3390/jpm11040301 33920764 PMC8071105

[B80] Laselva O. Conese M. (2022). Elexacaftor/tezacaftor/ivacaftor accelerates wound repair in cystic fibrosis airway epithelium. J. Pers. Med. 12, 1577. 10.3390/jpm12101577 36294716 PMC9605106

[B81] Laselva O. Molinski S. Casavola V. Bear C. E. (2018). Correctors of the major cystic fibrosis mutant interact through membrane-spanning domains. Mol. Pharmacol. 93, 612–618. 10.1124/mol.118.111799 29618585

[B82] Lee T. Sawicki G. S. Altenburg J. Millar S. J. Geiger J. M. Jennings M. T. (2022). Effect of Elexacaftor/Tezacaftor/Ivacaftor on annual rate of lung function decline in people with cystic fibrosis. J. Cyst. Fibros. 27:S1569–S1993. 10.1016/j.jcf.2022.12.009 36581485

[B83] Leonhardt K. Autry E. B. Kuhn R. J. Wurth M. A. (2021). CFTR modulator drug desensitization: Preserving the hope of long term improvement. Pediatr. Pulmonol. 56, 2546–2552. 10.1002/ppul.25437 33913624

[B84] Liessi N. Pesce E. Braccia C. Bertozzi S. M. Giraudo A. Bandiera T. (2020). Distinctive lipid signatures of bronchial epithelial cells associated with cystic fibrosis drugs, including Trikafta. JCI Insight 5, e138722. 10.1172/jci.insight.138722 32673287 PMC7455125

[B85] Loo T. W. Clarke D. M. (2017). Corrector VX-809 promotes interactions between cytoplasmic loop one and the first nucleotide-binding domain of CFTR. Biochem. Pharmacol. 136, 24–31. 10.1016/j.bcp.2017.03.020 28366727

[B86] Lopez A. Daly C. Vega-Hernandez G. MacGregor G. Rubi J. L. (2023). Elexacaftor/tezacaftor/ivacaftor projected survival and long-term health outcomes in people with cystic fibrosis homozygous for F508del. J. Cyst. Fibros. 10.1016/j.jcf.2023.02.004 36849331

[B87] Lowry S. Mogayzel P. J. Oshima K. Karnsakul W. (2022). Drug-induced liver injury from elexacaftor/ivacaftor/tezacaftor. J. Cyst. Fibros. 21, e99–e101. 10.1016/j.jcf.2021.07.001 34275759

[B88] Loyd I. Papac N. Hirshburg J. Levin J. Dannelley J. Dorris J. (2022). If at first you don't succeed, trikafta again. J. Pediatr. Pharmacol. Ther. 27, 467–469. 10.5863/1551-6776-27.5.467 35845559 PMC9268110

[B89] Ludovico A. Moran O. Baroni D. (2022). Modulator combination improves *in vitro* the microrheological properties of the airway surface liquid of cystic fibrosis airway epithelia. Int. J. Mol. Sci. 23, 11396. 10.3390/ijms231911396 36232697 PMC9569604

[B90] Mainz J. G. Zagoya C. Polte L. Naehrlich L. Sasse L. Eickmeier O. (2022). Elexacaftor-tezacaftor-ivacaftor treatment reduces abdominal symptoms in cystic fibrosis-early results obtained with the CF-specific CFabd-score. Front. Pharmacol. 13, 877118. 10.3389/fphar.2022.877118 35721187 PMC9203829

[B91] Martin C. Burnet E. Ronayette-Preira A. de Carli P. Martin J. Delmas L. (2021). Patient perspectives following initiation of elexacaftor-tezacaftor-ivacaftor in people with cystic fibrosis and advanced lung disease. Respir. Med. Res. 80, 100829. 10.1016/j.resmer.2021.100829 34091202

[B92] Martin C. Reynaud-Gaubert M. Hamidfar R. Durieu I. Murris-Espin M. Danner-Boucher I. (2022). Sustained effectiveness of elexacaftor-tezacaftor-ivacaftor in lung transplant candidates with cystic fibrosis. J. Cyst. Fibros. 21, 489–496. 10.1016/j.jcf.2022.01.012 35123901

[B93] Martiniano S. L. Nick J. A. Daley C. L. (2022). Nontuberculous mycobacterial infections in cystic fibrosis. Clin. Chest Med. 43, 697–716. 10.1016/j.ccm.2022.06.010 36344075

[B94] Mayer-Hamblett N. Nichols D. P. Odem-Davis K. Riekert K. A. Sawicki G. S. Donaldson S. H. (2021). Evaluating the impact of stopping chronic therapies after modulator drug therapy in cystic fibrosis: The SIMPLIFY clinical trial study design. Ann. Am. Thorac. Soc. 18, 1397–1405. 10.1513/AnnalsATS.202010-1336SD 33465316 PMC8513667

[B95] Mayer-Hamblett N. Ratjen F. Russell R. Donaldson S. H. Riekert K. A. Sawicki G. S. (2022). Discontinuation versus continuation of hypertonic saline or dornase alfa in modulator treated people with cystic fibrosis (SIMPLIFY): Results from two parallel, multicentre, open-label, randomised, controlled, non-inferiority trials. Lancet Respir. Med. S2213-2600 (22), 00434–00439. 10.1016/S2213-2600(22)00434-9 PMC10065895 36343646

[B96] McKinzie C. J. Doligalski C. T. Lobritto S. J. Coakley R. D. Gower W. A. (2022). Use of elexacaftor/tezacaftor/ivacaftor in liver transplant patients with cystic fibrosis. J. Cyst. Fibros. 21, 227–229. 10.1016/j.jcf.2021.07.017 34384711

[B97] Mederos-Luis E. González-Pérez R. Poza-Guedes P. Álava-Cruz C. Matheu V. Sánchez-Machín I. (2022). Toxic epidermal necrolysis induced by cystic fibrosis transmembrane conductance regulator modulators. Contact Dermat. 86, 224–225. 10.1111/cod.14002 34731505

[B98] Mehta Z. Kamal K. M. Miller R. Covvey J. R. Giannetti V. (2021). Adherence to cystic fibrosis transmembrane conductance regulator (CFTR) modulators: Analysis of a national specialty pharmacy database. J. Drug Assess. 10, 62–67. 10.1080/21556660.2021.1912352 33968464 PMC8078929

[B99] Middleton P. G. Mall M. A. Dřevínek P. Lands L. C. McKone E. F. Polineni D. (2019). Elexacaftor-Tezacaftor-Ivacaftor for Cystic Fibrosis with a Single Phe508del Allele. N. Engl. J. Med. 381, 1809–1819. 10.1056/NEJMoa1908639 31697873 PMC7282384

[B100] Miller A. C. Harris L. M. Cavanaugh J. E. Abou Alaiwa M. Stoltz D. A. Hornick D. B. (2022). The rapid reduction of infection-related visits and antibiotic use among people with cystic fibrosis after starting elexacaftor-tezacaftor- ivacaftor. Clin. Infect. Dis. 75, 1115–1122. 10.1093/cid/ciac117 35142340 PMC9525072

[B101] Miller M. J. Foroozan R. (2022). Papilledema and hypervitaminosis A after elexacaftor/tezacaftor/ivacaftor for cystic fibrosis. Can. J. Ophthalmol. 57, e6–e10. 10.1016/j.jcjo.2021.04.018 34058144

[B102] Molinski S. V. Shahani V. M. Subramanian A. S. MacKinnon S. S. Woollard G. Laforet M. (2018). Comprehensive mapping of cystic fibrosis mutations to CFTR protein identifies mutation clusters and molecular docking predicts corrector binding site. Proteins 86, 833–843. 10.1002/prot.25496 29569753

[B103] Morrison C. B. Shaffer K. M. Araba K. C. Markovetz M. R. Wykoff J. A. Quinney N. L. (2022). Treatment of cystic fibrosis airway cells with CFTR modulators reverses aberrant mucus properties *via* hydration. Eur. Respir. J. 59, 2100185. 10.1183/13993003.00185-2021 34172469 PMC8859811

[B104] Muirhead C. Verzasconi D. Joshi S. (2022). At-home compounding preparation of slow desensitization of elexacaftor/tezacaftor/ivacaftor for delayed hypersensitivity rash. Pediatr. Pulmonol. 57, 1779–1781. 10.1002/ppul.25938 35451238

[B105] Nichols D. P. Donaldson S. H. Frederick C. A. Freedman S. D. Gelfond D. Hoffman L. R. (2021). Promise: Working with the CF community to understand emerging clinical and research needs for those treated with highly effective CFTR modulator therapy. J. Cyst. Fibros. 20, 205–212. 10.1016/j.jcf.2021.02.003 33619012 PMC8686210

[B106] Nichols D. P. Paynter A. C. Heltshe S. L. Donaldson S. H. Frederick C. A. Freedman S. D. (2022). Clinical effectiveness of elexacaftor/tezacaftor/ivacaftor in people with cystic fibrosis: A clinical trial. Am. J. Respir. Crit. Care Med. 205, 529–539. 10.1164/rccm.202108-1986OC 34784492 PMC8906485

[B107] Nuytten A. Prevotat A. Le Rouzic O. Dekemp J. Gilliot S. Gautier S. (2022). Pulmonary hemorrhage in a neonate born to a woman with cystic fibrosis treated with targeted cystic fibrosis transmembrane conductance regulator modulator elexacaftor-tezacaftor-ivacaftor during pregnancy. Therapie 77, 743–745. 10.1016/j.therap.2022.01.020 35606190

[B108] O'Connor K. E. Goodwin D. L. NeSmith A. Garcia B. Mingora C. Ladores S. L. (2021). Elexacafator/tezacaftor/ivacaftor resolves subfertility in females with CF: A two center case series. J. Cyst. Fibros. 20, 399–401. 10.1016/j.jcf.2020.12.011 33353860 PMC9101452

[B109] Okiyoneda T. Veit G. Dekkers J. F. Bagdany M. Soya N. Xu H. (2013). Mechanism-based corrector combination restores ΔF508-CFTR folding and function. Nat. Chem. Biol. 9, 444–454. 10.1038/nchembio.1253 23666117 PMC3840170

[B110] Okroglic L. Sohier P. Martin C. Lheure C. Franck N. Honoré I. (2023). Acneiform eruption following elexacaftor-tezacaftor-ivacaftor treatment in patients with cystic fibrosis. JAMA Dermatol 159, 68–72. 10.1001/jamadermatol.2022.5208 36449298 PMC9713678

[B111] Pallenberg S. T. Held I. Minso R. Hansen G. Tümmler B. Dittrich A.-M. (2023). Differential effects of ELX/TEZ/IVA on organ-specific CFTR function in two patients with the rare CFTR splice mutations c.273+1G>A and c.165-2A>G. Front. Pharmacol. 14. 10.3389/fphar.2023.1153656 PMC10083416 37050906

[B112] Pallenberg S. T. Junge S. Ringshausen F. C. Sauer-Heilborn A. Hansen G. Dittrich A. M. (2022a). CFTR modulation with elexacaftor-tezacaftor-ivacaftor in people with cystic fibrosis assessed by the β-adrenergic sweat rate assay. J. Cyst. Fibros. 21, 442–447. 10.1016/j.jcf.2021.10.005 34756683

[B113] Pallenberg S. T. Pust M. M. Rosenboom I. Hansen G. Wiehlmann L. Dittrich A. M. (2022b). Impact of elexacaftor/tezacaftor/ivacaftor therapy on the cystic fibrosis airway microbial metagenome. Microbiol. Spectr. 10, e0145422. 10.1128/spectrum.01454-22 36154176 PMC9602284

[B114] Petersen M. C. Begnel L. Wallendorf M. Litvin M. (2022). Effect of elexacaftor-tezacaftor-ivacaftor on body weight and metabolic parameters in adults with cystic fibrosis. J. Cyst. Fibros. 21, 265–271. 10.1016/j.jcf.2021.11.012 34862121 PMC9999463

[B115] Piona C. Mozzillo E. Tosco A. Volpi S. Rosanio F. M. Cimbalo C. (2022). Impact of CFTR modulators on beta-cell function in children and young adults with cystic fibrosis. J. Clin. Med. 11, 4149. 10.3390/jcm11144149 35887914 PMC9319690

[B116] Prajapati B. B. Filippi A. Sears E. H. (2021). Chronic joint pain in a young adult with cystic fibrosis. Cureus 13, e17229. 10.7759/cureus.17229 34540456 PMC8442824

[B117] Ragan H. Autry E. Bomersback T. Hewlett J. Kormelink L. Safirstein J. (2022). The use of elexacaftor/tezacaftor/ivacaftor in patients with cystic fibrosis post liver transplant: A case series. Pediatr. Pulmonol. 57, 411–417. 10.1002/ppul.25779 34850610

[B118] Ramos K. J. Guimbellot J. S. Valapour M. Bartlett L. E. Wai T. H. Goss C. H. (2022). Use of elexacaftor/tezacaftor/ivacaftor among cystic fibrosis lung transplant recipients. J. Cyst. Fibros. 21, 745–752. 10.1016/j.jcf.2022.04.009 35474016 PMC9509406

[B119] Ren H. Y. Grove D. E. De La Rosa O. Houck S. A. Sopha P. Van Goor F. (2013). VX-809 corrects folding defects in cystic fibrosis transmembrane conductance regulator protein through action on membrane-spanning domain 1. Mol. Biol. Cell. 24, 3016–3024. 10.1091/mbc.E13-05-0240 23924900 PMC3784376

[B120] Reyes-Ortega F. Qiu F. Schneider-Futschik E. K. (2020). Multiple reaction monitoring mass spectrometry for the drug monitoring of ivacaftor, tezacaftor, and elexacaftor treatment response in cystic fibrosis: A high-throughput method. ACS Pharmacol. Transl. Sci. 3, 987–996. 10.1021/acsptsci.0c00103 33073196 PMC7551731

[B121] Ringshausen F. C. Sauer-Heilborn A. Büttner T. Dittrich A. M. Schwerk N. Ius F. (2023). Lung transplantation for end-stage cystic fibrosis before and after the availability of elexacaftor-tezacaftor-ivacaftor, Germany, 2012-2021. Eur. Respir. J. 61, 2201402. 10.1183/13993003.01402-2022 36517178

[B122] Roehmel J. F. Ogese M. O. Rohrbach A. Mall M. A. Naisbitt D. J. (2021). Drug allergy to CFTR modulator therapy associated with lumacaftor-specific CD4^+^ T lymphocytes. J. Allergy Clin. Immunol. 147, 753–756. 10.1016/j.jaci.2020.05.041 32526307

[B123] Rotolo S. M. Duehlmeyer S. Slack S. M. Jacobs H. R. Heckman B. (2020). Testicular pain following initiation of elexacaftor/tezacaftor/ivacaftor in males with cystic fibrosis. J. Cyst. Fibros. 19, e39–e41. 10.1016/j.jcf.2020.04.017 32471772

[B124] Ryan K. J. Guimbellot J. S. Dowell A. E. Reed-Walker K. D. Kerstner-Wood C. D. Anderson J. D. (2022). Quantitation of cystic fibrosis triple combination therapy, elexacaftor/tezacaftor/ivacaftor, in human plasma and cellular lysate. J. Chromatogr. B Anal. Technol. Biomed. Life Sci. 1213, 123518. 10.1016/j.jchromb.2022.123518 PMC12451711 36371965

[B125] Safirstein J. Grant J. J. Clausen E. Savant D. Dezube R. Hong G. (2021). Biliary disease and cholecystectomy after initiation of elexacaftor/ivacaftor/tezacaftor in adults with cystic fibrosis. J. Cyst. Fibros. 20, 506–510. 10.1016/j.jcf.2020.07.014 32736949

[B126] Salehi M. Iqbal M. Dube A. AlJoudeh A. Edenborough F. (2021). Delayed hepatic necrosis in a cystic fibrosis patient taking Elexacaftor/Tezacaftor/Ivacaftor (Kaftrio). Respir. Med. Case Rep. 34, 101553. 10.1016/j.rmcr.2021.101553 34815934 PMC8593462

[B127] Schnell A. Jüngert J. Klett D. Hober H. Kaiser N. Ruppel R. (2023). Increase of liver stiffness and altered bile acid metabolism after triple CFTR modulator initiation in children and young adults with cystic fibrosis. Liver Int. 10.1111/liv.15544 36797990

[B128] Schwarzenberg S. J. Vu P. T. Skalland M. Hoffman L. R. Pope C. Gelfond D. (2022). Elexacaftor/tezacaftor/ivacaftor and gastrointestinal outcomes in cystic fibrosis: Report of promise-GI. J. Cyst. Fibros. S1569-1993 (22), 01384–4. 10.1016/j.jcf.2022.10.003 PMC10144072 36280527

[B129] Scully K. J. Marchetti P. Sawicki G. S. Uluer A. Cernadas M. Cagnina R. E. (2022). The effect of elexacaftor/tezacaftor/ivacaftor (ETI) on glycemia in adults with cystic fibrosis. J. Cyst. Fibros. 21, 258–263. 10.1016/j.jcf.2021.09.001 34531155 PMC8918034

[B130] Shakir S. Echevarria C. Doe S. Brodlie M. Ward C. Bourke S. J. (2022). Elexacaftor-Tezacaftor-Ivacaftor improve Gastro-Oesophageal reflux and Sinonasal symptoms in advanced cystic fibrosis. J. Cyst. Fibros. 21, 807–810. 10.1016/j.jcf.2022.06.003 35718668

[B131] Shaughnessy C. A. Yadav S. Bratcher P. E. Zeitlin P. L. (2022b). Receptor-mediated activation of CFTR via prostaglandin signaling pathways in the airway. Am. J. Physiol. Lung Cell. Mol. Physiol. 322, L305–L314. 10.1152/ajplung.00388.2021 35020527 PMC8858663

[B132] Shaughnessy C. A. Zeitlin P. L. Bratcher P. E. (2021). Elexacaftor is a CFTR potentiator and acts synergistically with ivacaftor during acute and chronic treatment. Sci. Rep. 11, 19810. 10.1038/s41598-021-99184-1 34615919 PMC8494914

[B133] Shaughnessy C. A. Zeitlin P. L. Bratcher P. E. (2022a). Net benefit of ivacaftor during prolonged tezacaftor/elexacaftor exposure *in vitro* . J. Cyst. Fibros. 21, 637–643. 10.1016/j.jcf.2022.02.011 35248469 PMC9329187

[B134] Sheikh S. Britt R. D. Jr. Ryan-Wenger N. A. Khan A. Q. Lewis B. W. Gushue C. (2023). Impact of elexacaftor-tezacaftor-ivacaftor on bacterial colonization and inflammatory responses in cystic fibrosis. Pediatr. Pulmonol. 58, 825–833. 10.1002/ppul.26261 36444736 PMC9957929

[B135] Sondo E. Cresta F. Pastorino C. Tomati V. Capurro V. Pesce E. (2022). The L467F-F508del Complex Allele Hampers Pharmacological Rescue of Mutant CFTR by Elexacaftor/Tezacaftor/Ivacaftor in Cystic Fibrosis Patients: The Value of the *ex vivo* Nasal Epithelial Model to Address Non-Responders to CFTR-Modulating Drugs. Int. J. Mol. Sci. 23, 3175. 10.3390/ijms23063175 35328596 PMC8952007

[B136] Sosinski L. M. Martin C. H. Neugebauer K. A. Ghuneim L. J. Guzior D. V. Castillo-Bahena A. (2022). A restructuring of microbiome niche space is associated with Elexacaftor-Tezacaftor-Ivacaftor therapy in the cystic fibrosis lung. J. Cyst. Fibros. 21, 996–1005. 10.1016/j.jcf.2021.11.003 34824018 PMC9124239

[B137] Spoletini G. Gillgrass L. Pollard K. Shaw N. Williams E. Etherington C. (2022). Dose adjustments of Elexacaftor/Tezacaftor/Ivacaftor in response to mental health side effects in adults with cystic fibrosis. J. Cyst. Fibros. 21, 1061–1065. 10.1016/j.jcf.2022.05.001 35585012

[B138] Stanke F. Pallenberg S. T. Tamm S. Hedtfeld S. Eichhorn E. M. Minso R. (2023). Changes in cystic fibrosis transmembrane conductance regulator protein expression prior to and during elexacaftor-tezacaftor-ivacaftor therapy. Front. Pharmacol. 14, 1114584. 10.3389/fphar.2023.1114584.2023.1114584 36778025 PMC9911415

[B139] Stapleton A. L. Kimple A. J. Goralski J. L. Nouraie S. M. Branstetter B. F. Shaffer A. D. (2022). Elexacaftor-Tezacaftor- Ivacaftor improves sinonasal outcomes in cystic fibrosis. J. Cyst. Fibros. 21, 792–799. 10.1016/j.jcf.2022.03.002 35300931 PMC9470769

[B140] Steinack C. Ernst M. Beuschlein F. Hage R. Roeder M. Schuurmans M. M. (2023) Improved glucose tolerance after initiation of Elexacaftor/Tezacaftor/Ivacaftor in adults with cystic fibrosis. J. Cyst. Fibros. S1569-1993(23) 00006–11. 10.1016/j.jcf.2023.01.004 36669960

[B141] Streibel C. Willers C. C. Pusterla O. Bauman G. Stranzinger E. Brabandt B. (2023). Effects of elexacaftor/tezacaftor/ivacaftor therapy in children with cystic fibrosis - a comprehensive assessment using lung clearance index, spirometry, and functional and structural lung MRI. J. Cyst. Fibros. 2023 (22), 01432–1441. Epub ahead of print. 10.1016/j.jcf.2022.12.012 36635199

[B142] Stylemans D. Darquenne C. Schuermans D. Verbanck S. Vanderhelst E. (2022). Peripheral lung effect of elexacaftor/tezacaftor/ivacaftor in adult cystic fibrosis. J. Cyst. Fibros. 21, 160–163. 10.1016/j.jcf.2021.03.016 33832855

[B143] Szentpetery S. Foil K. Hendrix S. Gray S. Mingora C. Head B. (2022). A case report of CFTR modulator administration via carrier mother to treat meconium ileus in a F508del homozygous fetus. J. Cyst. Fibros. 21, 721–724. 10.1016/j.jcf.2022.04.005 35422395

[B144] Taylor-Cousar J. L. Jain R. (2021). Maternal and fetal outcomes following elexacaftor-tezacaftor-ivacaftor use during pregnancy and lactation. J. Cyst. Fibros. 20, 402–406. 10.1016/j.jcf.2021.03.006 33762125

[B145] Terlizzi V. Colangelo C. Marsicovetere G. D'Andria M. Francalanci M. Innocenti D. (2021). Effectiveness of Elexacaftor/Tezacaftor/Ivacaftor Therapy in Three Subjects with the Cystic Fibrosis Genotype Phe508del/Unknown and Advanced Lung Disease. GenesGenes (Basel) 12, 1178. 10.3390/genes12081178 PMC8391133 34440351

[B146] Tewkesbury D. H. Athwal V. Bright-Thomas R. J. Jones A. M. Barry P. J. (2023). Longitudinal effects of elexacaftor/tezacaftor/ivacaftor on liver tests at a large single adult cystic fibrosis centre. J. Cyst. Fibros.: S1569-1993(23) 00008–15. 10.1016/j.jcf.2023.01.007 36669962

[B147] Tomati V. Costa S. Capurro V. Pesce E. Pastorino C. Lena M. (2022). Rescue by elexacaftor-tezacaftor-ivacaftor of the G1244E cystic fibrosis mutation's stability and gating defects are dependent on cell background. J. Cyst. Fibros. S1569-1993 (22), 01425–1434. Epub ahead of print. 10.1016/j.jcf.2022.12.005 36543707

[B148] Tsai A. Wu S. P. Haseltine E. Kumar S. Moskowitz S. M. Panorchan P. (2020). Physiologically based pharmacokinetic modeling of CFTR modulation in people with cystic fibrosis transitioning from mono or dual regimens to triple-combination elexacaftor/tezacaftor/ivacaftor. Pulm. Ther. 6, 275–286. 10.1007/s41030-020-00124-7 32734574 PMC7672136

[B149] Tümmler B. (Editor) (2022). Mutation-specific therapies in cystic fibrosis (Bremen: UNI-MED).

[B150] Uluer A. Z. MacGregor G. Azevedo P. Indihar V. Keating C. Mall M. A. (2023). Safety and efficacy of vanzacaftor-tezacaftor-deutivacaftor in adults with cystic fibrosis: Randomised, double-blind, controlled, phase 2 trials. Lancet Respir. Med. 2023 (22), 00504–00505. 10.1016/S2213-2600(22)00504-5 PMC12815409 36842446

[B151] van Barneveld A. Stanke F. Claass A. Ballmann M. Tümmler B. (2008). CFTR protein analysis of splice site mutation 2789+5 G-A. J. Cyst. Fibros. 7, 165–167. 10.1016/j.jcf.2007.07.007 17707141

[B152] Van Goor F. Hadida S. Grootenhuis P. D. Burton B. Cao D. Neuberger T. (2009). Rescue of CF airway epithelial cell function *in vitro* by a CFTR potentiator, VX-770. Proc. Natl. Acad. Sc.i U. S. A. 106, 18825–18830. 10.1073/pnas.0904709106 PMC2773991 19846789

[B153] Van Goor F. Hadida S. Grootenhuis P. D. Burton B. Stack J. H. Straley K. S. (2011). Correction of the F508del-CFTR protein processing defect *in vitro* by the investigational drug VX-809. Proc. Natl. Acad. Sci. U. S. A. 108, 18843–18848. 10.1073/pnas.1105787108 21976485 PMC3219147

[B154] Veit G. Avramescu R. G. Perdomo D. Phuan P. W. Bagdany M. Apaja P. M. (2014). Some gating potentiators, including VX-770, diminish ΔF508-CFTR functional expression. Sci. Transl. Med. 6, 246ra97. 10.1126/scitranslmed.3008889 PMC4467693 25101887

[B155] Veit G. Roldan A. Hancock M. A. Da Fonte D. F. Xu H. Hussein M. (2020). Allosteric folding correction of F508del and rare CFTR mutants by elexacaftor-tezacaftor-ivacaftor (Trikafta) combination. JCI Insight 5, e139983. 10.1172/jci.insight.139983 32853178 PMC7526550

[B156] Veit G. Vaccarin C. Lukacs G. L. (2021a). Elexacaftor co-potentiates the activity of F508del and gating mutants of CFTR. J. Cyst. Fibros. 20, 895–898. 10.1016/j.jcf.2021.03.011 33775603 PMC8463622

[B157] Veit G. Velkov T. Xu H. Vadeboncoeur N. Bilodeau L. Matouk E. (2021b). A precision medicine approach to optimize modulator therapy for rare CFTR folding mutants. J. Pers. Med. 11, 643. 10.3390/jpm11070643 34357110 PMC8307171

[B158] Veit G. Xu H. Dreano E. Avramescu R. G. Bagdany M. Beitel L. K. (2018). Structure-guided combination therapy to potently improve the function of mutant CFTRs. Nat. Med. 24, 1732–1742. 10.1038/s41591-018-0200-x 30297908 PMC6301090

[B159] Walter E. Bass J. L. (2022). The effect of elexacaftor/tezacaftor/ivacaftor on hospitalizations and intravenous antibiotic use. Perm. J. 26, 73–79. 10.7812/TPP/21.089 35609157 PMC9126543

[B160] Welsner M. Schulte T. Dietz-Terjung S. Weinreich G. Stehling F. Taube C. (2022). Effect of triple combination CFTR modulator therapy on sleep in adult patients with cystic fibrosis. Respiration 101, 766–774. 10.1159/000524773 35598598

[B161] Westhölter D. Schumacher F. Wülfinghoff N. Sutharsan S. Strassburg S. Kleuser B. (2022). CFTR modulator therapy alters plasma sphingolipid profiles in people with cystic fibrosis. J. Cyst. Fibros. 21, 713–720. 10.1016/j.jcf.2022.02.005 35168870

[B162] Wisniewski B. L. Aylward S. C. Jordan C. O. Kopp B. T. Paul G. R. (2022). Hypervitaminosis A with fulminant secondary intracranial hypertension following personalized medicine-based Elexacaftor/Tezacaftor/Ivacaftor initiation in a preadolescent with cystic fibrosis. J. Cyst. Fibros. 21, e217–e220. 10.1016/j.jcf.2022.01.010 35131172

[B163] Wright B. A. Ketchen N. K. Rasmussen L. N. Bartels A. R. Singh S. B. (2022). Impact of elexacaftor/tezacaftor/ivacaftor on vitamin D absorption in cystic fibrosis patients. Pediatr. Pulmonol. 57, 655–657. 10.1002/ppul.25781 34859619

[B164] Zhang L. Albon D. Jones M. Bruschwein H. (2022a). Impact of elexacaftor/tezacaftor/ivacaftor on depression and anxiety in cystic fibrosis. Ther. Adv. Respir. Dis. 16, 17534666221144211. 10.1177/17534666221144211 36562554 PMC9793010

[B165] Zhang L. Shrestha C. L. Robledo-Avila F. Jaganathan D. Wisniewski B. L. Brown N. (2022b). Cystic fibrosis macrophage function and clinical outcomes after elexacaftor/tezacaftor/ivacaftor. Eur. Respir. J. 2022, 2102861. Epub ahead of print. 10.1183/13993003.02861-2021 PMC10066828 36265882

